# Transcription regulation by RNA-induced structural strain in duplex DNA

**DOI:** 10.1093/nar/gkaf429

**Published:** 2025-05-22

**Authors:** Aura Cencini, Graziano Rilievo, Alessandro Cecconello, Federica Tonolo, Massimiliano Babbucci, Enrico Negrisolo, Massimiliano Magro, Fabio Vianello

**Affiliations:** Department of Comparative Biomedicine and Food Science (BCA), University of Padova, Campus Agripolis, viale dell’Università 16, 35020 Legnaro (PD), Italy; Department of Comparative Biomedicine and Food Science (BCA), University of Padova, Campus Agripolis, viale dell’Università 16, 35020 Legnaro (PD), Italy; Department of Comparative Biomedicine and Food Science (BCA), University of Padova, Campus Agripolis, viale dell’Università 16, 35020 Legnaro (PD), Italy; Department of Molecular and Translational Medicine (DMMT), University of Brescia, viale Europa 11, 25135 Brescia (BS), Italy; Department of Comparative Biomedicine and Food Science (BCA), University of Padova, Campus Agripolis, viale dell’Università 16, 35020 Legnaro (PD), Italy; Department of Comparative Biomedicine and Food Science (BCA), University of Padova, Campus Agripolis, viale dell’Università 16, 35020 Legnaro (PD), Italy; Department of Comparative Biomedicine and Food Science (BCA), University of Padova, Campus Agripolis, viale dell’Università 16, 35020 Legnaro (PD), Italy; Department of Agronomy, Food, Natural Resources, Animals, and Environment (DAFNAE), University of Padova, Campus Agripolis, viale dell’Università 16, 35020 Legnaro (PD), Italy; Department of Comparative Biomedicine and Food Science (BCA), University of Padova, Campus Agripolis, viale dell’Università 16, 35020 Legnaro (PD), Italy; Department of Comparative Biomedicine and Food Science (BCA), University of Padova, Campus Agripolis, viale dell’Università 16, 35020 Legnaro (PD), Italy

## Abstract

Non-coding RNAs belong to a heterogenous family that, among other functions, acts as a biomolecular regulator of gene expression. In particular, lncRNAs, which are estimated to be as numerous as coding RNAs in humans, are thought to interact with genomic DNA to form triple helices. However, experimental evidence of their involvement with processes, such as chromatin structure dynamics or RNA transcription, is still missing. Here, a mechanism of transcription enhancement/inhibition is described, where hybrid RNA-DNA triplexes regulate transcription rates in *Escherichia coli* promoter-based designed architectures. Sequences associated with triplexes were identified in a library of bacterial promoters and characterized *in vitro*, followed by a synthetic biology approach to verify their ability to control transcription and translation. A model of the triplex-promoter complex was produced showing that transcription enhancement is due to a distortion of the duplex DNA as a consequence of its conjugation with RNA in the triplex assembly. These results point at a mechanism of RNA function that is still unknown and could be common in more complex organisms, such as metazoans including mammals, where non-coding RNAs are more abundant and are believed to play a fundamental role in determining hetero/euchromatin and transcription modulation.

## Graphical Abstract

**Graphical Abstract:**
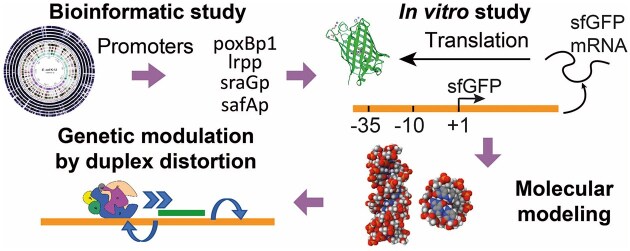

## Introduction

From the study of RNA as messenger RNAs (mRNAs), the investigation of its biological role in cells experienced enormous developments. The study of nucleic acid secondary structures, a rather recent investigation approach, boosted the molecular biology research field describing in detail the structures and functions of ribonucleoproteins, riboswitches, and ribozymes [1–6]. Nowadays, theoretical tools and experimental protocols allow the production of designed nucleic acid nanostructures and the realization of orthogonal biosynthetic or metabolic paths in cells [7–13]. Due to the need of integrating many different disciplines to study such structures, the biology field has seen the birth of specific interdisciplinary scientific branches. Indeed, synthetic biology, along with DNA and RNA nanotechnology studies, contributed to clarify how nucleic acid nanostructures, such as geometrical shapes, nano-mechanical devices, and smart materials, can be built by following simple base-pairing rules [14, 15]. Specifically, a single nucleic acid can assemble in solution into double-, triple-, or quadruple-geometries following Watson–Crick and Hoogsteen rules [16]. Multiple-strand nanostructures are also possible, for example, by origami or tiling methods [17–19], and they were used as biosynthetic nanomachines and scaffolds for the ordered assembly of proteins and nanoparticles, as well as molecular circuits [20–23].

Since the nineties of the last century, triple-strand nucleic acid structures, also called triplexes, were experimentally demonstrated to form *in vitro* and to inhibit transcription in a specific scenario where RNA interacts with genomic DNA [24–28]. Nevertheless, experimental research struggled to extract detailed information from this phenomenon during the following years, mainly due to the high cost to produce the starting material, the perishable nature of RNA, and the difficulty in delivering it into the cell [29]. In spite of this, hybrid triplex structures are again under the spotlight since they were recently proposed to play a role as functional structures of long non-coding RNAs in human cells and, particularly, in the organization of chromatin architecture and transcription regulation [30–35]. New *in vitro* studies by our group showed that binding of a triplex-forming oligonucleotide (TFO) RNA to a duplex DNA triplex target site (TTS) in a synthetic transcription unit (TU), could either enhance or inhibit transcription as a consequence of the target gene or triplex geometry, namely depending on its structure and position within the promoter [36–38]. This new effect associated with triplex formation fueled the idea that triplexes could fully regulate transcription in cells by the direct interaction of non-coding RNAs with duplex DNA, in the absence of a protein mediator. In the present study, we show that bacterial sequences can form triplexes *in vitro* and can regulate transcription and translation in cell extracts. One of the final goals is to determine the mechanisms behind the regulation effect.

**Figure 1.**
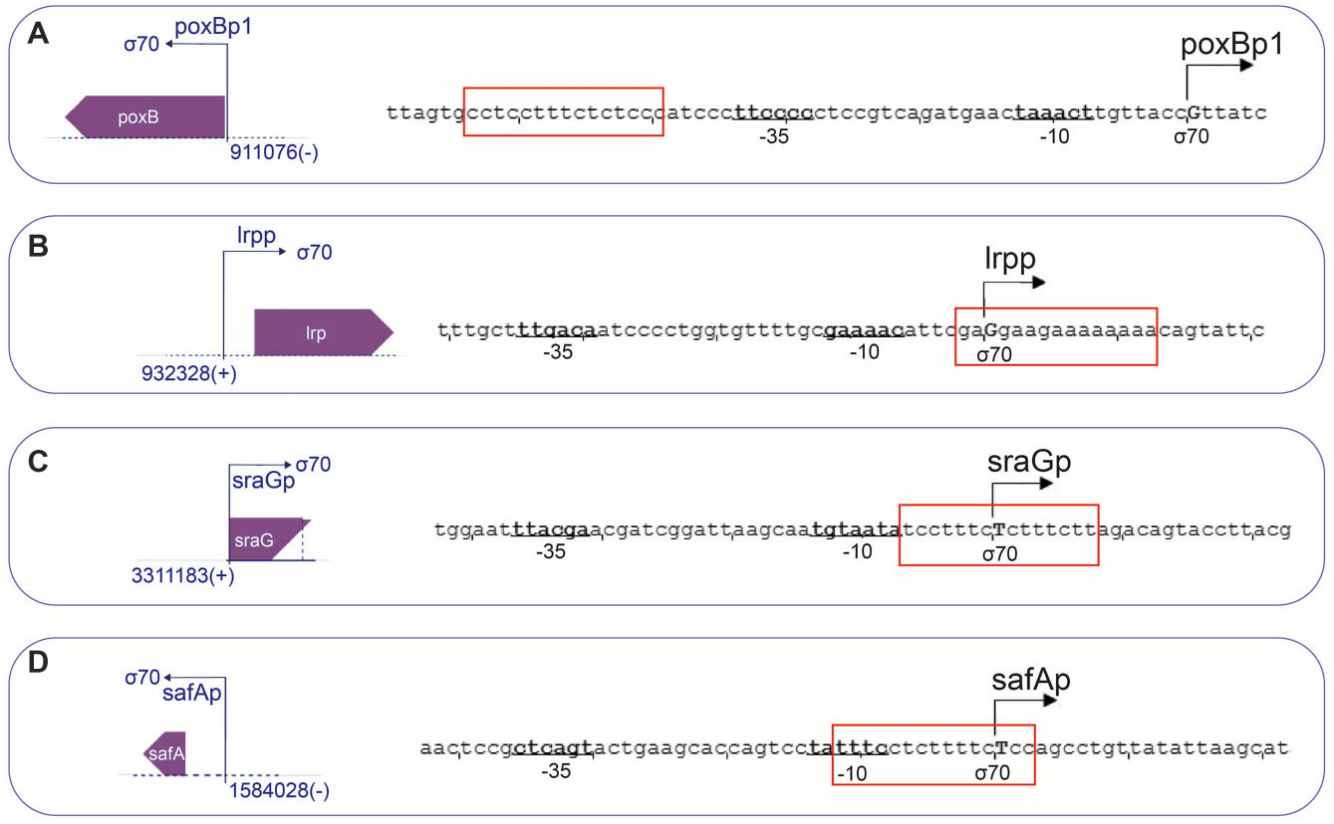
On the left, schemes of the TUs associated with the promoters identified by *Triplexator*: (**A**) poxB promoter 1 (poxBp1) and its associated coding sequence for pyruvate oxidase; (**B**) lrp promoter (lrpp) associated with coding sequence for DNA-binding transcriptional dual regulator Lrp, leucine-responsive regulatory protein; (**C**) sraG promoter (sraGp) associated with coding sequence for small regulatory RNA; (**D**) safA promoter (safAp) associated with coding sequence for two-component system connector SafA. On the right, promoter structures corresponding to TUs reported on the left, where the −35/−10 consensus is marked by underlined nucleotides, +1 position is marked by an angled arrow reporting the promoter name, and the TTS is marked with a red square.

The genome of *Escherichia coli* K12 strain was screened for putative TTSs that fitted our established model for triplex-mediated transcription regulation, using an untargeted method (i.e. not sequence directed). Such sequences were then integrated within an artificial TU architecture and tested for *in vitro* transcription in the presence of the corresponding TFO. Shortly, the TTS had to be at least 15 base pair long, located in close proximity to the −35 and −10 σ^70^ consensus, and containing a polypurinic sequence. For this task, we used *Triplexator* [39], a well-known triplex-scavenger software, on *E. coli* K12 promoter library (for additional details on the constraints and software, please see “Materials and methods” and Supplementary Information). Indeed, a number of TTS-containing promoters were found to match our searching criteria, from where four were selected for further characterization: lrpp, sraGp, poxBp1, and safAp. The structures of these promoters and their associated TUs are schematically depicted in Fig. 1 (details on the schemes, software, and database used for the generation of bioinformatic data are reported in the Supplementary Information, including Supplementary Table S1 showing software results and associated scores).

Schemes in Fig. 1 A–D, on the left, show the binding site for the RNAp/σ^70^ (black angled arrow) to genomic DNA (dotted black line), a purple area labeled with the gene name (poxB, lrp, sraG, or safA), and the genomic position in the (+) or (−) filament as a number. Schemes on the right side show the promoter sequence comprising underlined −35/−10 consensus and +1 nucleotide marked with an angled black arrow, while red boxes identify the TTSs.

Synthetic sequences corresponding to the four TTSs and eight TFOs (a purine-pyrimidine pair for each TTS) were used for the thermodynamic analysis of the parameters associated with triplex formation using electrophoretic mobility shift assay (EMSA) and melting temperature profiling. These sequences were then used to assemble TUs to produce, in the presence of the appropriate TFO, the fluorogenic aptamer Broccoli *in vitro*, demonstrating that these sequences behave according to the inhibition/enhancement rules of the aforementioned triplex geometry model [36, 37]. Finally, these structures were assembled in a transcription-translation (TX-TL) system where they regulated the expression of green fluorescent protein (GFP) in bacterial lysates.

## Materials and methods

### Sample preparation

All oligonucleotides were purchased from IDT (Integrated DNA Technologies, USA) as synthetic products, in the freeze-dried powder form, dissolved in ultrapure water (resistivity at least 18.0 MΩ⋅cm) and used without additional treatments (further details, including all DNA and RNA sequences for the initial thermodynamic characterization, are reported in the SI, Supplementary Tables S2–S6). Double-strand DNA sequences were allowed to anneal in a thermal cycler by adding 10 μM of each complementary strand in 50 mM NaCl, with a temperature ramp from 95°C to 10°C. Stock solutions were then stored at −20°C until use. Samples for EMSA, temperature-dependent denaturation experiments, and RNA-polymerization rates, were prepared in a modified version of the commercial RNA-polymerization buffer (from here on: modified RNAp reaction buffer) by New England BioLabs (NEB, USA). The buffer included 40 mM Tris–HCl, 150 mM NaCl, 30 mM MgCl_2_, 1 mM dithiothreitol (DTT), and 0.01% Triton X-100, at pH 6.9 (see SI for a complete list of materials and manufacturers).

### Electrophoretic separations

Triplex formation between the double-strand DNA and the purine/pyrimidine TFO was carried out by adding 100 nM of the different TTSs in the presence of increasing concentrations (from 1 nM to 50 μM) of the relative purinic or pyrimidinic TFO, in the modified RNAp reaction buffer. EMSA experiments were conducted in 12% acrylamide/bis-acrylamide gels (19:1) prepared in 1X tris-borate-EDTA buffer at pH 6.9, containing 10 mM MgCl_2_, 1.25 mg mL^−1^ ammonium persulfate, and 0.05% tetramethylethylenediamine. The gel loading dye was prepared with 50% glycerol in water and 0.1% bromophenol blue. Electrophoresis runs were carried out at 100 V for 2 h in an ice-cooled water bath. Gels were then stained in Oxazole Gold 1X staining solution, and emission of the bands was acquired through UV excitation using an iBright1500 (ThermoFisher Scientific) imaging system. All experiments were conducted at least in triplicates and results are summarized in Supplementary Table S7.

### Triplex melting curves

Temperature-dependent denaturation analyses were performed using 100 nM of the different TTSs in the presence of increasing concentrations of the relative purinic or pyrimidinic TFO (from 1 nM to 100 μM), in the modified RNAp reaction buffer, and averaging data of at least three independent experiments. After an initial incubation at 4°C for 30 min, 10 μL of the mixture was transferred to a tube with 8 μL of water and 2 μL of Thiazole Green 1X solution. Triplex denaturation was performed in a Stratagene Mx3000P quantitative-polymerase chain reaction (qPCR) instrument (Agilent Technologies, USA), measuring fluorescence emission while performing a temperature ramp from 25°C to 95°C. Results were expressed as the negative first derivative of the fluorescence signal.

### *In vitro* RNA polymerization rate analysis

100 nM of the DNA duplex templates were incubated with either the purinic or the pyrimidinic relative TFO, at different concentrations (template design and additional experimental details are reported in the SI, sequences are listed in Supplementary Table S8). A 30-min incubation at 4°C was carried out in the modified RNAp reaction buffer at pH 6.9. The polymerization reaction was then started with the addition of 16 U mL^−1^ of RNAp (σ70-saturated *E. coli* RNAp holoenzyme, NEB, USA) and 4 mM rNTPs. To follow Broccoli RNA aptamer formation, DFHBI-1T (Lucerna technologies, USA) was also added at a final concentration equal to 160 μM. Fluorescence emission was recorded with a Victor X4 plate reader (Perkin Elmer, USA) equipped with excitation/emission filters 390/535 nm, respectively, and using Lumox 384-well plates (Sarstedt, Germany). All RNA-polymerization experiments were conducted at 29°C for 8 h. Fluorescence emission data for rate analysis was limited to a time interval of 120 min (ca. 1 h after the start), where reaction reached stability and experimental points were fitted linearly using Origin software (OriginLab Corporation, USA). Fluorescence rates were expressed as averaged fluorescence emission intensity changes over time of at least three independent experiments.

### *In vitro* transcription-translation experiments

The duplex DNA sequence coding for super-folder Green Fluorescent Protein (sfGFP) was amplified by PCR using three sets of primers, each containing a specific *E. coli* promoter. The forward (FWD) primer sequence included the bacterial TTS of interest and the ribosome binding site sequence. The reverse (RVS) primer sequence for sfGFP was identical for all templates, and it contained the stop sequence for polymerization (see also Supplementary Fig. S1, SI, for the sequence design and Supplementary Table S8 for a list of DNA sequences). The amplification was carried by mixing One-Taq Master Mix 1X (NEB, USA), 200 nM of the primer set, and 0.5 nM of the sfGFP template, in a 20-cycle PCR. A quality assessment of the amplification was performed by electrophoresis of the products (see Supplementary Fig. S2, SI). Around 50 nM of the obtained template were then mixed with 500 nM of the corresponding TFO, in the modified RNAp reaction buffer, and samples were incubated at +4°C for 30 min to promote triplex formation. To start the *in vitro* transcription-translation process, 5 μL of the triplex-containing sample were mixed with 3 μL of NEBExpress S30 Synthesis exctract (NEB, USA), protein synthesis buffer 0.5X (NEB, USA), and 400 U mL^−1^ of murine RNase inhibitor (NEB, USA). sfGFP fluorescence emission was recorded with a Victor X4 plate reader at 29°C for 8 h. Fluorescence signals were then analyzed with Origin software in a 2-h time interval. From this analysis, averaged linear rates of sfGFP production from at least three independent experiments were obtained as fluorescence intensity changes in time and compared across the different samples.

### SDS-PAGE and western blot

To determine the end-point variations in sfGFP levels upon *in vitro* transcription-translation experiments and triplex modulation, immunoblot experiments were performed. Proteins were separated by SDS-PAGE (12%) and subjected to Western Blot analysis using Bio-Rad Trans-Blot Turbo Transfer System (see also SI for additional experimental details). The membrane was saturated overnight at 4°C using 3% BSA buffer (0.05 M Tris, 0.15 M NaCl, and 3% BSA, at a pH of 7.5), first probed with anti-sfGFP primary antibody (PA5-109258, Thermofisher Scientific, Waltham, USA) for 2.5 h and then with goat anti-rabbit IgG (H + L) secondary antibody, 1:10 000 (31 460, ThermoFisher Scientific, Waltham, USA) in 1% BSA, 0.05 M Tris, 0.15 M NaCl, and 0.01% sodium azide, at a pH of 7.5. Signal detection was performed using the ECL system (Clarity Western ECL Substrate, BIORAD, Hercules, USA) with iBright1500 imaging system. Results from at least three independent experiments were averaged and represented as bar plots.

### Molecular modeling

3D-NuS on-line software (https://iith.ac.in/3dnus/Triplex.html) was used to produce the DNA-DNA-RNA triplex structures as pdb files. The generated structures were then analyzed using Mol* 3D Viewer (https://www.rcsb.org/3d-view) that allowed measurement of the average distance between pairs of nucleotides (selection mode > measurements > add > distance). Plots of average distances against triplex modulation were then generated using the Origin software.

## Results

### Target screening and triplex thermodynamic stability

To test the hybrid triplex geometry model on actual genomic targets, TTSs were initially identified in a model bacterial genome (*E. coli* K12) using publicly available *Triplexator* software. A list containing 81-nt-long sequences centred at all known σ^70^ promoters was screened according to parameters specified in the “Materials and methods” section and the Supplementary Information. The selected sequences, along with extended schematic representations of the associated loci, are reported in the Supplementary Information and specifically in Supplementary Table S2 and Supplementary Fig. S3, and they were named lrpp, poxBp1, safAp, and sraGp. TFOs were designed to form purine- or pyrimidine-motif triplexes (i.e. TFOs containing AG or UC only bases, respectively), where the ssRNA forms Hoogsteen interactions with the polypurine-containing DNA strand of the duplex, in an antiparallel or parallel orientation, respectively. For this reason, each target was tested for the formation of the two types of triplex. TFO, TTS sequences, and a schematic representation of the triplex geometries are reported in Supplementary Tables S3 and S4 and Supplementary Fig. S4.

**Figure 2.**
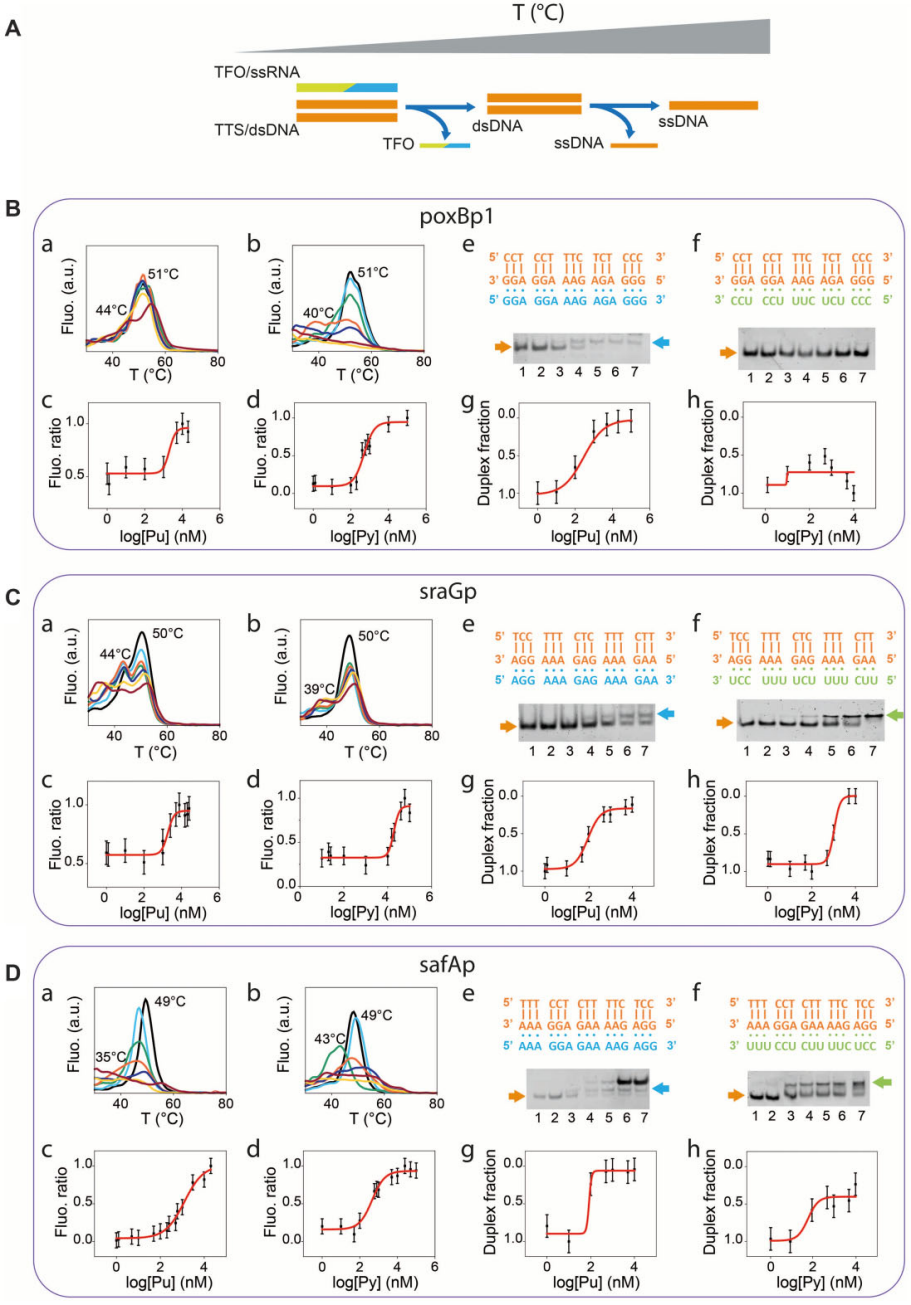
(**A**) Scheme of the triplex and duplex structure sequential denaturation at increasing temperatures. (**B–** **D**): (a and b) Experimental melting temperature analysis varying the concentration of the purinic (Pu) or pyriminidic (Py) TFO, respectively. Colored lines light blue, green, orange, dark blue, yellow, and red refer to increasing TFO concentration. TFO concentrations are as follows: in B, a- 0, 1, 5, 10, 10^2^, 5 · 10^2^, and 5 · 10^3^ nM; b- 0, 10, 10^2^, 4 · 10^2^, 10^3^, 5 · 10^3^, and 10^4^ nM; in C, a- 0, 2.5, 5, 7.5, 10, 15, and 20 μM; b- 0, 5, 10, 20, 40, 60, and 80 μM; in D, a- 0, 1, 50, 10^2^, 2.5 · 10^2^, 10^3^, and 10^4^ nM; b- 0, 0.01, 0.1, 1, 10, 25, and 50 μM. (c and d) Sigmoidal fits of the RNA and DNA temperature dissociation peak ratios for increasing concentrations of purinic (Pu) and pyrimidinic (Py) TFO, respectively. (e and f) Sequences (top) and EMSA (bottom) of poxBp1, sraGp, and safAp TTSs with individually analyzed TFOs, either purinic, in e, or pyrimidinic, in f. Orange, blue, and green arrows identify DNA duplexes, Pu triplex, and Py triplex, respectively. Lane numbers mark different TFO concentrations, with 1 identifying the smallest one. TTS concentration was fixed at 100 nM, while TFO concentration was varied as follows: in B, e- 0, 1, 10^2^, 5 · 10^2^, 10^3^, 5 · 10^3^, and 10^4^ nM; f- 0, 10, 10^2^, 5 · 10^2^, 10^3^, 5 · 10^3^, and 10^4^; in C, e- 0, 1, 10, 50, 10^2^, 5 · 10^2^, and 10^3^ nM; f- 0, 10, 50, 10^2^, 5 · 10^2^, 10^3^, and 5 · 10^3^ nM; in D, e- 0, 10, 10^2^, 5 · 10^2^, 10^3^, 5 · 10^3^, and 10^4^; f- 0, 10, 10^2^, 5 · 10^2^, 10^3^, 5 · 10^3^, and 10^4^ nM. (g and h) Sigmoidal fits of the duplex band normalized intensity for increasing concentrations of purinic (Pu) and pyrimidinic (Py) TFO, respectively. Each experiment was performed in three replicates. Data points represent mean values and error bars indicate standard deviations.

In both methods, i.e. temperature-dependent dissociation (Tm) analysis and EMSA, samples were prepared containing a constant duplex TTS concentration while exposing it to the corresponding TFO in a range of concentrations. Regarding temperature-dependent dissociation analysis, a scheme of the sequential dissociation events is depicted in Fig. 2A, while experimental results are summarized in Fig. 2 B–D, a–d panels. The data showed a peak at ca. 50°C and a second peak at lower temperatures, and these were attributed to the duplex and the triplex dissociation events, respectively. The triplex dissociation peak (i.e. the temperature at which the triplex is at 50% its maximum concentration) was found in the interval from 39°C to 44°C, depending on triplex motif and composition. Samples showing only one Tm peak were interpreted as incapable of producing a stable triplex and were not analyzed further (i.e. lrpp did not show a triplex-associated peak and experimental results are reported in the Supplementary Information, Supplementary Fig. S5. In addition, a representative set of experiments with one-mismatch comprising sequences were conducted, Supplementary Table S5 and Supplementary Fig. S6, showing no triplex formation). The intensity ratio of the two peaks was then plotted against the TFO concentration and fitted with a sigmoid. Fig. 2 reports experimental data as follows: poxBp1, Panel B; sraGp, Panel C; and safAp, Panel D. TFO concentration at the symmetry point of the sigmoid fit corresponds to an estimate of the dissociation constant of the duplex-triplex equilibrium, providing information on the stability of the triplex complex.

Next, we performed EMSA experiments on each TTS sequence with its corresponding purinic or pyrimidinic TFO at increasing concentrations (Fig. 2 B–D, e–h). Typically, the separation shows bands corresponding to the duplex DNA TTS (orange arrow), the purine or pyrimidine triplex band (blue or green arrow, respectively) and TFO excess as a third band at higher molecular weights, which only becomes apparent since micromolar concentrations of SybrGold bind to RNA as well. The appearance of a slower band at high TFO concentrations and the simultaneous weakening of the duplex band intensity were interpreted as an indication of triplex formation, while experimental set-ups that did not show such behavior were interpreted as incapable of producing a stable triplex (similarly to melting curve analysis, lrpp did not show triplex formation and those data were reported in the Supplementary Information, Supplementary Fig. S7). To avoid band misinterpretation, a series of samples were incubated with RNase H, as control experiments. Although DNA-RNA duplexes should not form, due to the absence of complementary Watson–Crick sequences, their presence was ruled out experimentally by using the specific ribonuclease that digests the RNA part in a hybrid DNA-RNA duplex. Results of electrophoretic separations of the control samples are reported in Supplementary Fig. S8 (see also Supplementary Table S6 for the control sequences), showing no difference across samples and confirming the previous band attribution.

Band intensities of Fig. 2 EMSA gels were plotted against TFO concentration showing a sigmoidal data distribution in line with melting curve analyses. In these conditions, dissociation constants, *K*_d_ values, could be estimated as they are equal to the TFO concentration when duplex and triplex are equimolar (for the set of assumptions under which this could be applied, please see Supplementary Information). These values were collectively reported in Supplementary Table S7 (see also Supplementary Fig. S9, Supplementary Information, for additional experimental points and sigmoid fittings of EMSA triplex band intensities). Although the fluorescent probe affinity effect introduced bias in the estimates, not allowing a direct comparison of EMSA and Tm dissociation constants, gel electrophoresis results further corroborated the occurrence of triplexes. Most importantly, band intensities plotted against TFO concentration showed a sigmoidal data distribution in line with melting curve analyses, confirming that RNA-DNA binding responds to common equilibrium principles.

This first part, substantiating the RNA-DNA conjugation in a wide range of the explored *E. coli* genomic targets, provides the molecular basis to move forward with the investigation of triplex geometry-related effects. Results of this initial biophysical analysis are summarized in Table 1. It should be noted that, while still being well-fitted by a sigmoid, *K*_d_ measurements obtained from EMSA experiments tend to underestimate dissociation constant values in our experimental setup and should be treated with caution (Supplementary Table S7, SI). This aspect is probably due to the gel separation process, being rather different with respect to formation/dissociation of complexes in solution, where there is no gel matrix nor electrophoretic migration affecting thermodynamic stability. Therefore, the proposed approach, based on melting temperature analysis in solution, was selected as a more adequate method to estimate *K*_d_ values.

**Table 1.** Triplex dissociation constants obtained from Tm analyses reported in Fig. 2

TTS	TFO	Tm *K*_d_ (μM)
safAp	Pu	1.2 ± 0.2
	Py	0.45 ± 0.06
sraGp	Pu	2 ± 1
	Py	19 ± 1
poxBp1	Pu	2.0 ± 0.2
	Py	0.5 ± 0.1

Temperature-dependent dissociation events produced *K*_d_ values in the 0.5–19 μM interval, with pyrimidine triplexes characterized by more extreme values, while purine triplex dissociation constants seem less erratic with values in the 1–2 μM range. This behavior seems to point at a sequence dependency more relevant for pyrimidine triplexes than for purine triplexes, probably due to known competing secondary structures, i.e. quadruplexes.

### Triplex-regulated *in vitro* transcription of Broccoli

**Figure 3.**
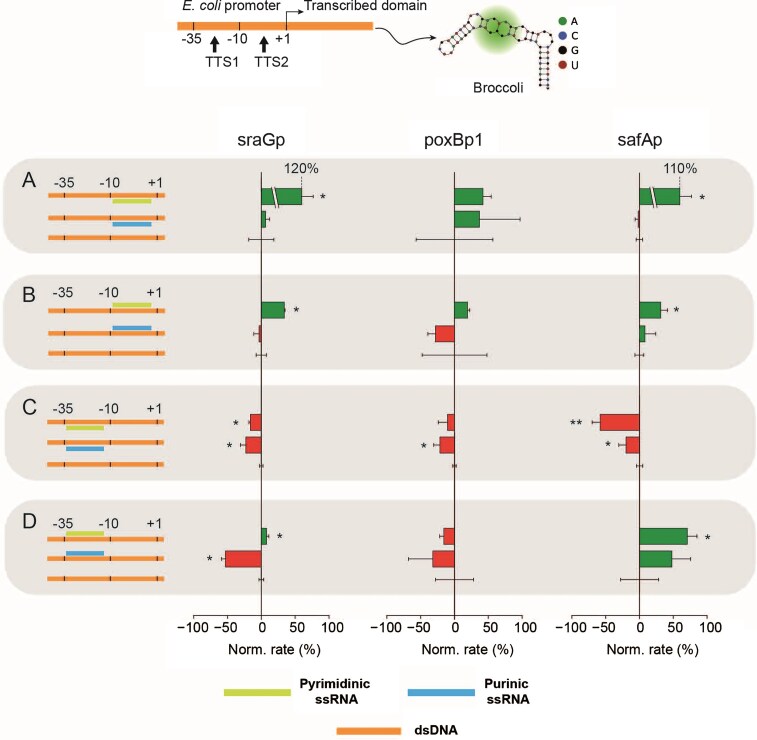
Broccoli transcription modulation using *E. coli*-based TUs and respective TFOs. The top scheme depicts the TU used for *in vitro* transcription experiments, where TTS1 and TTS2 indicate the two possible domains in which the TTSs for the different analyzed promoter (sraGp, poxBp1, and safAp) were inserted; (**A**) TTS positioned downstream of the promoter consensus, with the polypurinic domain in the template strand; (**B**) TTS positioned downstream of the promoter consensus, with the polypurinic domain in the sense strand; (**C**) TTS positioned within the promoter consensus, with the polypurinic domain in the template strand; and (**D**) TTS positioned within the promoter consensus, with the polypurinic domain in the sense strand. The columns reporting the experimental results in bar graphs are associated with, from left to right, sraGp, poxBp1, and safAp. Bar plots of the fluorescence change rates, green or red bars, indicate enhancement or inhibition, respectively, and they refer to a reference sample of TX-TL without TFO (error bar associated with 0% modulation). The star symbols next to the bar plots represent the *P*-value level obtained from a t-test: * for *P* < 0.05 and ** for *P* < 0.01. Each experiment was performed in three replicates. Bars represent mean values (normalized with respect to the control) and error bars indicate standard deviations.

Triplex TTSs validated in the previous step were introduced in a set of artificial TUs producing the fluorogenic aptamer Broccoli, Fig. 3, top scheme. All these units shared a common structure: A promoter for σ^70^ RNAp (i.e. consensus −35/−10), upstream of the template for Broccoli, where two domains, one within the promoter consensus (TTS1) and one downstream of the consensus (TTS2), were used for placing a specific TTS sequence. Four geometries were tested, where the polypurine strand was either in the sense or antisense strand, between the −35 and −10 domains, or downstream of the −10 domain. This approach produced eight combinations of TTS positions (four geometries, *vide supra*) and two TFO compositions (i.e. either purine or pyrimidine motif). Simplified schemes of the TU promoter structures are represented on the left side of the respective experimental analysis in Fig. 3 A–D. For further details on the TU design and specific sequences, see Supplementary Information, Supplementary Table S8.

RNA polymerization was followed by fluorescence intensity increase due to the formation of the Broccoli-dye (i.e. DFHBI-1T) complex, which has a ca. 2000-fold quantum yield enhancement compared to the dye alone. The emission intensity change rate was used as an estimate of the transcription rate in the presence of either the purine or pyrimidine TFO (i.e. the same TFOs used in the biophysical triplexes analysis), while a sample without TFO was used as a reference. The linear rates from three independent experiments were collected, and averages are represented graphically in Fig. 3 as red bars for inhibition or green bars for enhancement, while a summary of all transcription modulation average values is reported in Table 2 (see also Supplementary Figs S10–S12 for representative fluorescence kinetics).

**Table 2.** Transcription modulation of sraGp, poxBp1, and safAp engineered TUs by triplex formation. TU templates are indicated by the letters A, B, C, and D and correspond to the schemes shown in Fig. 3

Template	TFO	Norm. Rate (%)
sraGp (A)	Pu	+6 ± 6
	Py	+120 ± 70
sraGp (B)	Pu	-3 ± 8
	Py	+34 ± 1
sraGp (C)	Pu	-23 ± 8
	Py	-16 ± 2
sraGp (D)	Pu	-53 ± 6
	Py	+8 ± 3
poxBp1 (A)	Pu	+40 ± 70
	Py	+40 ± 10
poxBp1 (B)	Pu	−30 ± 10
	Py	+19 ± 3
poxBp1 (C)	Pu	−21 ± 9
	Py	−10 ± 10
poxBp1 (D)	Pu	−30 ± 40
	Py	−16 ± 6
safAp (A)	Pu	−2 ± 4
	Py	+110 ± 30
safAp (B)	Pu	+10 ± 20
	Py	+30 ± 10
safAp (C)	Pu	−20 ± 10
	Py	−60 ± 10
safAp (D)	Pu	+50 ± 30
	Py	+70 ± 10

Results are in agreement with our previously reported system, [36, 37] where a triplex downstream of the promoter consensus resulted in transcription enhancement in three geometries out of four and showed inhibition in one case, Fig. 3 A and B. Symmetric results were observed when the TTS was placed within the promoter consensus showing inhibition with three geometries and one case of enhancement, Fig. 3 C and D. It should be noted that in a few cases the system did not show significative modulation compared to the reference, due to a relatively large standard deviation. This was attributed to a rather slow transcription rate resulting from weak promoters that hindered the possibility of estimating the modulation with sufficient accuracy. It is remarkable that no invalidation of the previous rule set emerged by applying the published triplex geometry model. Indeed, present results show inverted transcription modulation only in the case of the aforementioned inaccurate estimates. To conclude, out of the 24 inhibition/enhancement modulation behaviors shown in Fig. 3, none is in contradiction with our paradigm.

### GFP expression in *E. coli* extracts

**Figure 4.**
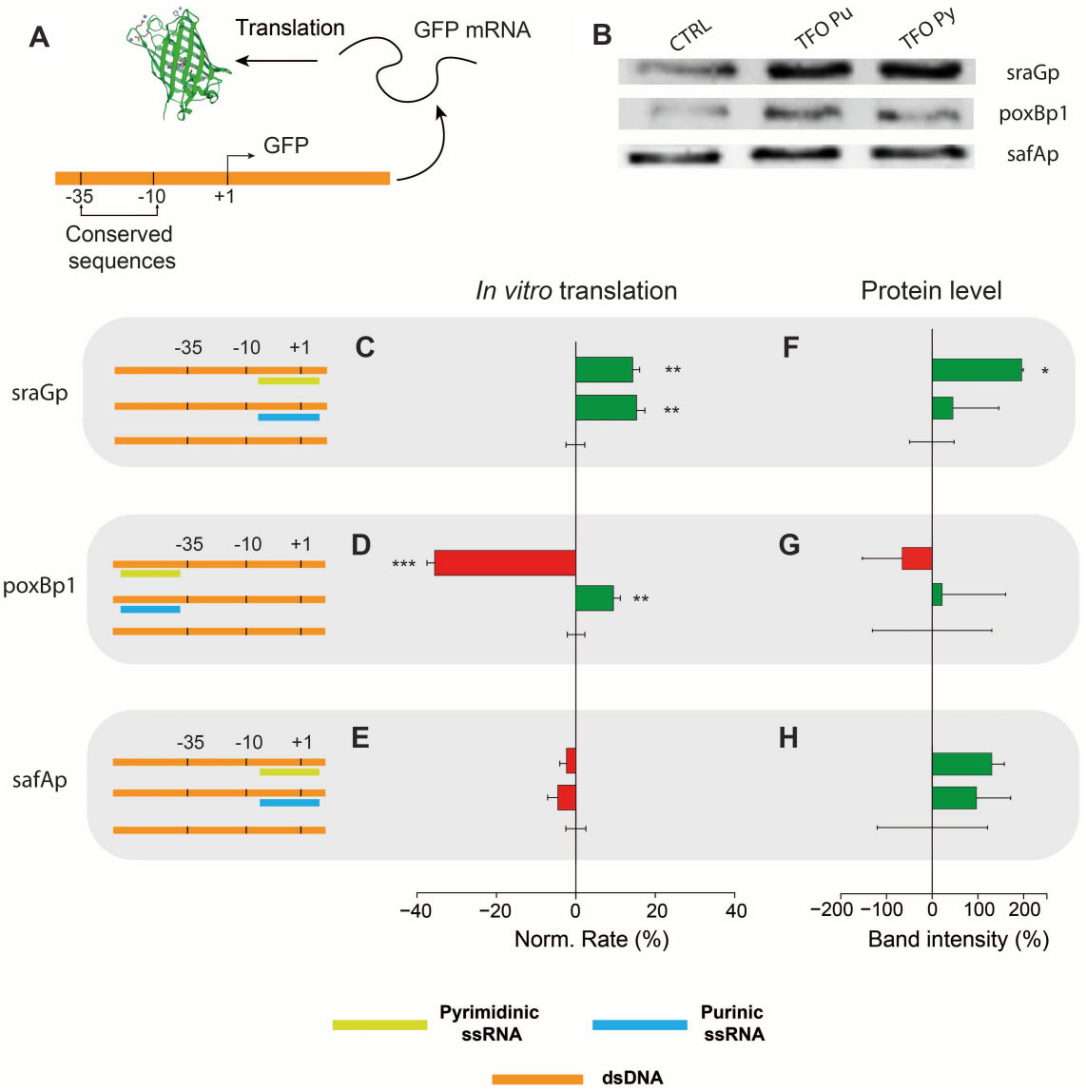
GFP production using triplex technology. (**A**) Scheme of the GFP transcription-translation system (TX-TL); (**B**) Bands corresponding to GFP immunoblot analysis, where CTRL corresponds to the reference TX-TL samples without TFO. (**C–E**) Modulation of GFP biosynthesis where the artificial TU depicted in Panel A was subjected to its associated TFO (C- sraGp, D- poxBp1, and E- safAp). On the left, schemes of the specific promoter where the color-coded TFO bar is positioned on top of (polypurinic sequence is in the sense strand) or under (polypurinic sequence is in the template strand) the dsDNA. On the right, bar plots of the fluorescence change rates (green or red bars indicate enhancement or inhibition, respectively) are referred to a reference sample of TX-TL without TFO (error bar at 0% modulation). (**F–H**) Bar plots of the band intensity relative to panel B, normalized to the TX-TL reference sample. The star symbols next to the bars represent the *P*-value level obtained from a t-test: * for *P* < 0.05, ** for *P* < 0.01, and *** for *P* < 0.001. Each experiment was performed in three replicates. Bars represent mean values (normalized with respect to the control) and error bars indicate standard deviation.

To provide evidence that supported the idea of the system working in cells, *E. coli* extracts were used to express a fluorescent protein. A TU architecture equivalent to the one used for *in vitro* transcription experiments was designed, containing the original *E. coli* promoters reported in Fig. 1 A–D, and controlling the template for super-folder GFP (sfGFP). A general scheme of the TU is reported in Fig. 4 A, while representative immunoblots and averaged experimental results relative to protein biosynthetic rates and quantification are shown in Fig. 4 B and Fig. 4 C-H, respectively. For further details on the TU design, specific sequences, and template quantification by gel electrophoresis see Supplementary Information, Supplementary Table S9, and Supplementary Figs S1 and S2.

Similarly to previous experiments, the transcription-translation (TX-TL) system was operated in the presence of the specific TU and micromolar concentrations of the respective TFO. sfGFP synthesis was monitored by its fluorescence emission and representative kinetics are reported in Supplementary Fig. S13. In this case, we observed a longer lag-time with respect to the transcription-only experiments. This was attributed to a retardation of the ribosomal activity that is dependent on a sufficient amount of sfGFP coding-RNA to accumulate in the reaction volume, which was estimated to be ca. 1 h. Considering this delay, a linear fitting of the translation rate was operated in a 1 hour-shifted time-window with respect to transcription-only experiments.

The fitted linear rates from three independent experiments were averaged, and TFO-containing samples were compared to reference samples containing no TFO. Results are reported in Fig. 4 C–E, as bar plots. Here, a clear difference could be observed, mirroring the results of the previous section. Specifically, three configurations showed enhancement (the highest modulation was equal to + 15%) and one configuration showed a significant inhibition (the strongest modulation was equal to −38%). In addition to protein biosynthetic rates, end-point estimates of sfGFP production levels were measured by immunoblot in the presence of the appropriate TFO, Fig. 4B (see the “Materials and methods” section and SI for additional technical details and immunoblot results, Supplementary Fig. S14 and Supplementary Table S10). Average sfGFP levels of three independent experiments are reported as bar plots in Fig. 4 F–H, showing results in line with the previous rate estimates, although less statistically significant. Overall, on the basis of the general set of rules mentioned above, the ability to effectively exert a control over protein biosynthesis, in an actual biological matrix by tailoring triplex assemblies, represents an exciting demonstration of the general feasibility of the proposed geometry principle.

### Molecular modeling of the triplex-containing promoter

**Figure 5.**
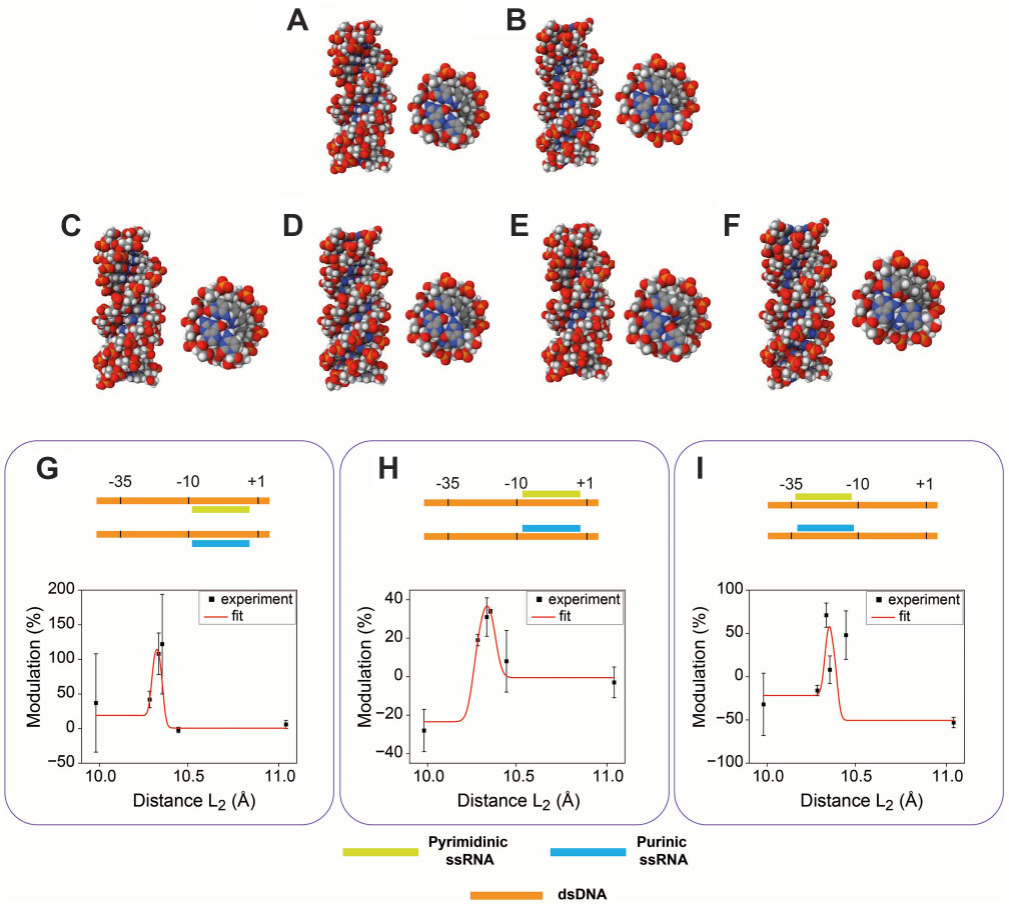
Molecular modeling of triplex formation according to each individual TTS-TFO pair. Side- and top-views of the respective pyrimidine and purine motif modeled triplexes for the TTSs found in the promoter: safAp (**A** and **B**), poxBp1 (**C** and **D**), and sraGp (**E** and **F**). **G**–**I**: Schemes of the triplex geometry (top side) and its associated average nucleotide distance at one end of the triplex model, reported in A–F, plotted against the respective transcription modulation.

To analyze geometric parameters associated with the formation of the triplexes *in silico*, we modeled the structures using 3D-NuS and the 15 nucleotide long TTS and TFO nucleic acid sequences. The algorithm produced eight pdb files that were rendered as space-filling representations in Fig. 5 A–F, as side- and top-views, referred to the central triple helix axis (for the pdb files, please see Online Content). Indeed, all six combinations of duplex DNA and single-strand RNA were correctly modeled, showing slightly different geometries for the purine and pyrimidine motifs. These models were then used for geometrical considerations.

In an attempt to describe the mechanism of triplex-regulated transcription enhancement, we tested the hypothesis that a distortion of the DNA duplex, caused by its association with the TFO via Hoogsteen interactions, could affect the geometry of the promoter region increasing the affinity of the RNAp/σ^70^ complex for the promoter. To do this, four geometric parameters were extracted from the models: Average distance between nucleotide pairs belonging to the same plane of the Watson–Crick/Hoogsteen interactions at either the 5′ or 3′ of the polypurinic sequence (L_1_ and L_2_, respectively), the difference between these two values (|L_1_-L_2_|), and the average length of the triple helix (L_3_). These measurements were reported in Table 3 (see also, Supplementary Figs S15 and S16 for representative inter-nucleotide distance schemes and additional measurements). Using transcription modulation percentages, as measured using the designed TUs comprising TTSs for inhibitory/enhancing triplexes, we then plotted the experimental rates associated with the respective triplex, placed in the same TU position, against individual geometric parameters. Quite surprisingly, a peak-like curve became evident for three of these series, as shown in Fig. 5 G–I, suggesting an optimum nucleotide distance for the highest enhancement. Moreover, all plots were in accordance on the same 10.3 Ångstrom peak-associated length.

**Table 3.** Average distances between: L_1_ and L_2_ (i.e. two nucleotides belonging to the same plane, at the 5′ or 3′ end of the triplex, respectively), L_1_-L_2_ difference, and L_3_ between the two planes at the 5′ and 3′ ends of the triplex (i.e. average length of the triple helix)

	poxBp1 Pu	poxBp1 Py	safAp Pu	safAp Py	sraGp Pu	sraGp Py
L_1_ (Å)	10.57	10.33	10.66	10.50	11.14	10.91
L_2_ (Å)	9.98	10.28	10.44	10.33	11.04	10.35
|L_1_-L_2_| (Å)	0.59	0.05	0.22	0.17	0.1	0.56
L_3_ (Å)	47.84	47.81	47.94	47.89	47.67	47.75

## Discussion

In this work, a *corpus* of experimental and theoretical results was assembled to support the idea that hybrid triplex complexes (where a ssRNA binds to duplex DNA) formed near or within a bacterial promoter, could regulate genes by accelerating or slowing the observed transcription rate in a predictable way. This was demonstrated first by characterizing thermodynamically the TTSs identified in strain K12 of *E. coli* genome (Fig. 2) and then monitoring their effect on transcription (Fig. 3). The two techniques, temperature-dependent dissociation and EMSA, were used to provide an initial evaluation of the triplex stability (Table 1 and Supplementary Table S7). Indeed, pyrimidine triplexes are generally more stable than purine ones due to the competition of the latter with quadruplex structures, while Tm-based analysis and EMSA-derived values are in accordance (although EMSA is generally underestimating *K*_d_ values), with the exception of pyrimidine poxBp1, showing analogous sigmoid behaviors and pointing at classic equilibrium paradigms (for further considerations see Supplementary Information). Lrpp TTS did not show any triplex formation in the presence of the appropriate TFO and was not further analyzed. This low stability was attributed to the triplex nucleotide content, being mostly adenines, and therefore lacking a sufficient sequence-complexity to promote triplex formation. It is worth noting that in a biological context, 15-nt-long triplexes should be considered as short units of probably longer tandem repeats, especially in organisms more complex than bacteria. As shown by other works [37, 40], these repeats would further stabilize the dsDNA-ssRNA association, strengthening, in turn, their effect on transcription modulation. Nevertheless, the short TTSs used in this study, along with TFO-matching sequences in the *E. coli* genome (see SI, Supplementary Table S11 for a list of best-match results) point at a possible regulation by short triplexes in simple organisms.

Transcription experiments in the presence of the appropriate TTS-containing TU and its respective purine- or pyrimidine-TFO were then conducted *in vitro*. To normalize the triplex effect and avoid promoter affinity differences, the TTSs were excised from their original *locus* and positioned in an artificial TU. Following this method, only the effect due to the triplex-forming portion was observed, while all other elements were assumed to have no differential effects across different TUs, due to their identical architecture besides the TTS. Indeed, up- or downregulation effects, shown in Fig. 3, were found to be in line with a recently proposed rational model [36, 37].

**Figure 6.**
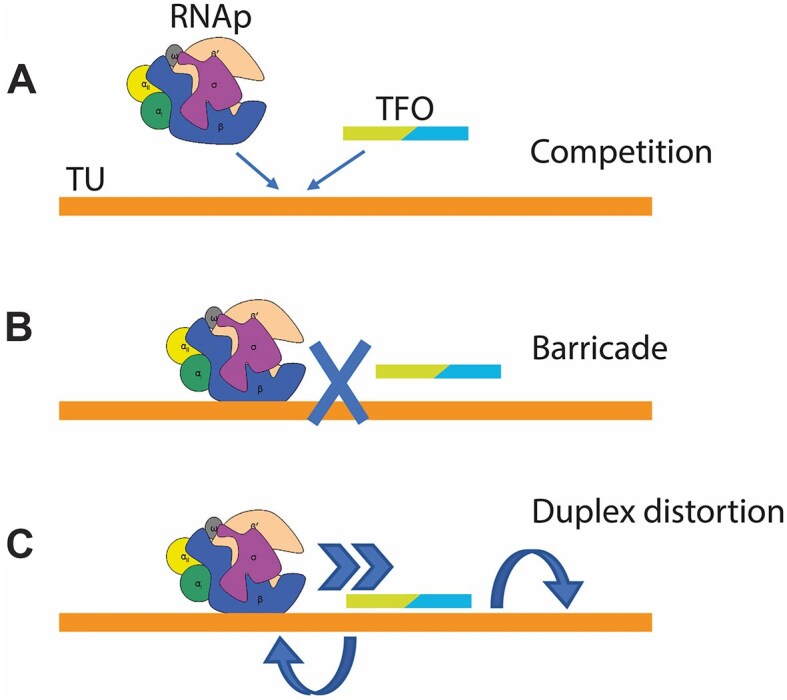
Schemes representing three different triplex-mediated mechanisms of transcription modulation: (**A**) Competition, (**B**) Barricade, and (**C**) triplex-induced duplex distortion. The double-colored TFO indicates that both motifs are possible.

We then tried to explore the effect of triplexes on protein levels by coupling *in vitro* transcription and translation. New constructs were designed, containing a promoter and the gene for sfGFP. The production of sfGFP, where its biosynthesis was controlled by the original promoters found in *E. coli*, was then monitored by fluorescence emissions. The TUs constructed by the fusion of sraGp, poxBp1, or safAp and sfGFP gene sequences were mixed with the appropriate TFO, and sfGFP biosynthetic rates were estimated by fluorescence emission changes. In addition, end-point sfGFP levels were quantified by immunoblot, showing results in line with transcription-translation rate estimates, in most cases (Fig. 4). Indeed, these results mirrored the transcription rate modulation and supported the idea that RNA can modulate gene expression by triplex formation within genomic targets. This can be explained by an alteration of the equilibrium RNAp_ON_/RNAp_OFF_ (i.e. the ratio between the actively polymerizing RNAp_ON_ and the inactive RNAp_OFF_) which is affected by the sigma factor-promoter complex strength. This strength, namely the affinity constant, was thought to be altered by the TFO due to two main mechanisms: Competition for the promoter target sequence or blockage of the RNAp by a “barricade effect”, Fig. 6. While these mechanisms would account for inhibitory effects, an explanation for the enhancement seems to be elusive. For this reason, an additional mechanism was considered: A distortion of the triplex flanking regions, that depends on triplex base-composition and geometry, generating an increased stability of the DNA-σ^70^/RNAp complex, and therefore an improved sigma factor/RNAp recruitment efficiency. It should be noted that in our experimental setup, there is only limited transcription re-initiation, therefore the triplex effect on this mechanism was not considered.

This TFO-induced duplex strain hypothesis was tested comparing geometric parameters extracted from the triplex molecular models and the associated transcription modulation. Indeed, a biphasic sigmoidal fitting identified an optimum for transcription enhancement at a duplex strain where average nucleotide distances are ca. 10.3 Å, supporting the idea that, while competition and RNAp blockage are likely responsible for the triplex inhibitory effects, duplex strain caused by triplex formation could explain the enhancement effects, mediated by affinity constant changes.

In conclusion, we demonstrated that 15-nt-TFOs could bind to the promoter region of a bacterial gene forming a triplex and modulate protein levels in a predictable way by up- or downregulating the respective RNA transcription rates. While transcription inhibition was a result of two already reported main mechanisms, that are RNAp blockage or competition with the triplex, the unprecedented up-modulation was attributed to a distortion in duplex geometry. These levels of fine regulation point at a yet unknown general genetic control that could be far more relevant in higher organisms (e.g. eukaryotic cells) where the role of regulating RNAs, e.g. lncRNAs, is mediated by nucleic acid secondary structures such as triplexes. In this context, a study on the conservation of triplex-promoter pairs and their horizontal transfer among different organisms would be pivotal since it could reveal evolutionary aspects. Hence, the presented triplex paradigm could be of crucial importance for its potential implications in human health, giving rise to a new landscape of opportunities and challenges, for instance in the context of triplexes as therapeutic targets. In this view, the study of nucleic acid secondary and tertiary structures [41, 42], as alternative dynamic geometries to the double helix, could revolutionize the current idea of gene therapy, integrating new strategies with current ones [43], for example peptide nucleic acids [44–46], based on the concept of RNA modulated transcription or the study of such structures as a source of genomic instability [47]. Clearly, the enticing vision of its bench-to-clinical translation deserves not just a mid-term research planning, but rather the involvement of a broad interdisciplinary network in the scientific community. In the next future, among others, three major issues will have to be coped with: (i) triplex-operated transcription regulation might be a general biological mechanism that need to be tested in other models, expanding the panel of constructs, and using advanced delivery systems in cells; (ii) although some studies proposed biomolecular components as stabilizers of nucleic acid secondary structures [28, 33], how such densely charged nano-biostructures can be stabilized in cells is still an open question that needs to be addressed, and (iii) the need for a high-throughput investigation of large triplex libraries including mismatching duplex-TFO sequences and their role in transcription regulation. Thus, the seminal content of this study is expected to boost the research on RNA secondary structures *in vivo* and strengthen the efforts for elucidating their role in genetic control.

## Supplementary Material

gkaf429_Supplemental_Files

## Data Availability

The data underlying this article are available in the article and in its online supplementary material.
