# Supplementary material for: Transcription regulation by RNA-induced structural strain in duplex DNA
Source: Nucleic Acids Res. 2025 May 22;53(10):gkaf429. doi: 10.1093/nar/gkaf429 (PMC12096072; doi:10.1093/nar/gkaf429)
Supplement: gkaf429_Supplemental_Files [file gkaf429_supplemental_files.zip › SI_clean_copy.docx]

**Transcription Regulation by RNA-Induced Structural Strain in Duplex DNA**

Aura Cencini,^1,ǂ^ Graziano Rilievo,^1,ǂ^ Alessandro Cecconello,^1,2,^* Federica Tonolo,^1^ Massimiliano Babbucci,^1^ Enrico Negrisolo,^1,3^ Massimiliano Magro,^1^ Fabio Vianello^1^

^1^ Department of Comparative Biomedicine and Food Science (BCA), University of Padova, Campus Agripolis, viale dell’Università 16, 35020, Legnaro (PD), Italy.

^2^ Department of Molecular and Translational Medicine (DMMT), University of Brescia, viale Europa 11, 25135, Brescia (BS), Italy

^3^ Department of Agronomy, Food, Natural Resources, Animals, and Environment (DAFNAE), University of Padova, Campus Agripolis, viale dell’Università 16, 35020, Legnaro (PD), Italy

^ǂ^These authors contributed equally to this work.

*correspondence can be addressed to A. Cecconello, email: alessandro.cecconello@unibs.it

**Supplementary Information**

**Contents**

[**1.** **Instruments** 3](#_Toc195617522)

[**2.** **Reagents** 3](#_Toc195617523)

[**3.** ***In Silico* prediction of RNA–DNA Triplexes** 4](#_Toc195617524)

[**4.** **TTS Sample preparation** 4](#_Toc195617525)

[**5.** **Electrophoretic Mobility Shift Assay** 5](#_Toc195617526)

[**6.** **Temperature-Dependent Triplex/Duplex Dissociation** 5](#_Toc195617527)

[**7.** ***In Vitro* Transcription** 5](#_Toc195617528)

[**8.** ***In Vitro* Transcription-Translation** 6](#_Toc195617529)

[**9.** **PCR product quantification** 7](#_Toc195617530)

[**10.** **Electrophoretic Mobility Shift Assay with RNase H** 9](#_Toc195617531)

[**11.** **SDS-PAGE and Western Blot** 9](#_Toc195617532)

[**12.** **DNA-RNA triplex modelling using 3D-NuS and extraction of structural distances** 10](#_Toc195617533)

[**13.** **Biphasic gaussian fitting of the triplex-induced distortion-modulation effect** 10](#_Toc195617534)

# **Instruments**

Double strand annealing procedure and PCR were performed with a Mastercycler Nexus-GX (Eppendorf Inc.). Time-dependent fluorescence measurements were performed using a VICTOR X4 2030 Multilabel Reader (Perkin Elmer, MA, USA) with 485/535 nm excitation/emission filters, along with Lumox 384 multiwell plates (Sarstedt, Germany). Melting temperature experiments were conducted using a Stratagene Mx3000P (Agilent Technologies) and samples were placed in tubes with optically clear caps (Thermo-Fisher Scientific Inc.). Electrophoresis runs were conducted in a Mini-PROTEAN Tetra System electrophoresis chamber (BIO-RAD), connected to a PowerEase Touch 350W power supply (Invitrogen, Thermo-Fisher Scientific Inc.). Western Blot was performed using a Trans-Blot Turbo Transfer System (BIO-RAD). Electrophoresis gel images and Western Blot band images were acquired with an iBright1500 imaging system (Invitrogen, Thermo-Fisher Scientific Inc.). Absorbance measurement were acquired with a Cary 60 (Agilent Technologies) UV-visible spectrophotometer. The data analysis software used to produce all the statistical and graphical analysis was OriginPro 2018b (OriginLab Corp.).

# **Reagents**

All reagents were purchased at the highest commercially available purity and were used without further treatments. All DNA and RNA sequences were ordered from IDT (Integrated DNA Technologies, USA) as freeze-dried material. MgCl_2_, Tris-borate-EDTA (TBE), acrylamide/bis-acrylamide solution, ammonium persulfate (APS), anti-sfGFP polyclonal primary antibody, Goat anti-rabbit IgG secondary antibody, and RNase H were purchased from ThermoFisher Scientific (USA). Sodium azide was purchased from Fluka (USA). Triton X-100, glycerol, bromophenol blue, bovine serum albumin (BSA), Tween 20, ColorBurst electrophoresis marker, and N,N,N’,N’-tetramethylethylenediamine (TEMED) were purchased from Sigma Aldrich/Merck (USA). NaCl was purchased from J. T. Baker (USA), and Tris was purchased from Carlo Erba (Italy). Oxazole Gold and Thiazole Green nucleic acid stains were purchased from Biotium (USA). Broccoli fluorescent ligand (Z)-4-(3,5-difluoro-4-hydroxybenzylidene)-2-methyl-1-(2,2,2-trifluoroethyl)-1H-imidazol-5(4H)-one (DFHBI-1T) was purchased from Lucerna (USA). σ70-saturated *E. coli* RNA polymerase (holoenzyme) and rNTPs solution were purchased from New England Biolabs (NEB, USA), as well as NEBExpress S30 synthesis extract, RNAse inhibitors, and One-Taq Master Mix. Clarity Western ECL Substrate was purchased from BIORAD (USA).

# ***In Silico* prediction of RNA–DNA Triplexes**

Potential triplex targeting sites (TTS) on the *E. coli* genome (GenBank: GCA_000005845.2) and the promoter sequences of the correlated mRNAs (RegulonDB database) were identified with *Triplexator[1]* software package, a computational framework for the *in silico* prediction of triplex structures. The TFO predictions were performed with the parameters -l 15 -e 3 -g 10 -m R/Y/M -dc 5 -fm 0 -of 0 -fr  (where l is the lower length bound, e is the error rate less than 3%, g is the guanine rate less than 10%, m is the type of triplex motif; R is a purine motif, Y is a pyrimidine motif, M is a purine–pyrimidine motif, fr denotes filter repeats, dc is the maximum number of duplication in the target genome and of is the output format). Primary results of the analysis are reported in **Table s1**.

| Duplex-ID | Start | End | Score | Strand | Guanine-rate | Duplicates | TTS | TTSs (abs) | TTSs (rel) |
| --- | --- | --- | --- | --- | --- | --- | --- | --- | --- |
| fadRp1 | 1 | 19 | 18 | - | 0.28 | -1 | AAAAAGAAGAAAAAGGGA | 10 | 0.0105 |
| groSp | 5 | 26 | 21 | - | 0.29 | -1 | AAGGGGGAAAAAGAAAAAAAA | 28 | 0.0294 |
| lolAp | 16 | 31 | 15 | - | 0.33 | -1 | GAGGAAAGAAGAAAA | 1 | 0.00105 |
| lrpp | 58 | 73 | 15 | + | 0.27 | -1 | GAGGAAGAAAAAAAA | 1 | 0.00105 |
| poxBp1 | 6 | 21 | 15 | - | 0.6 | -1 | GGGAGAGAAAGGAGG | 1 | 0.00105 |
| safAp | 48 | 63 | 15 | - | 0.4 | -1 | GGAGAAAAGAGGAAA | 1 | 0.00105 |
| sraGp | 53 | 68 | 15 | - | 0.33 | -1 | AAGAAAGAGAAAGGA | 1 | 0.00105 |

Table s1. Summary table of Triplexator analysis statistics. Duplex-ID: id of the double-stranded sequence providing the TTS. Start: start index of the feature. End: end index of the feature. Score: score of the feature/triplex (number of matches). Strand: strand of the duplex providing the poly-purine tract. Guanine-rate: proportion of guanines w/r/t the target able to participate in triplex formation. Duplicates: Number of times this triplex feature or respective target does occur in the corresponding sequence set not supported when searching for triplexes. TTSs (abs): absolute number of maximal TTSs found in the sequence. TTSs (rel): length-adjusted triplex potential.

# **TTS Sample preparation**

Double strand DNA structures were produced by preparing an aqueous solution containing 10µM of each strand and 50mM NaCl, incubating then the solution in the Mastercycler. The annealing steps were as follows: 95 °C for 5 minutes and a temperature ramp from 95°C to 25°C. dsDNA stock solutions were stored at -20°C and used when needed.

# **Electrophoretic Mobility Shift Assay**

EMSA experiments were carried out in a modified version of the recommended NEB buffer for RNA polymerization, containing 40Mm Tris-HCl, 150mM NaCl, 30mM MgCl_2_, 1mM DTT, 0.01% Triton X-100, at pH 6.9. 12% acrylamide/bis-acrylamide 29:1 gel was prepared following an established procedure in TBE 1x buffer, containing 10mM MgCl_2_. The gel was run at 100V (20 mA, 2 W) for 2 hours in an ice bath and then stained with Oxazole Gold. Gel pictures were acquired using Invitrogen iBright 1500 gel imager. Bands were analyzed using the gel imager software and the band intensities, given as Local Corrected Volume values, were fitted using Origin Lab software. In particular, the intensities of the triplex bands and of the duplex bands (y) were plotted against the logarithm of the corresponding TFO concentration (x). These experimental points were fitted using a dose-response function:

$$y=A1+\frac{A2-A1}{1+{10}^{(log{(x}_{0})-x)p}}$$

Where A1 and A2 are the left and right asymptotes, respectively, x_0_ is the TFO concentration at the inflection point (i.e., E_50_), and p is the slope of the sigmoid at the inflection point.

# **Temperature-Dependent Triplex/Duplex Dissociation**

Melting curves experiments were conducted using the same buffer described in the previous paragraph. The samples were prepared to contain 100nM of the double strand TTS and the concentration of the selected TFO was varied from one sample to another. After samples preparation, they were incubated for 30 minutes at +4°C, in order for the triplex to properly form. 10 µL of the samples, together with 8 µL of ultrapure water and 2 µL of thiazole green 20X, were transferred in qPCR white tubes. These were then placed in Stratagene Mx3000P qPCR machine, which performed a single cycle with 30 seconds at 25°C and a temperature ramp from 25°C to 95°C. Melting curves were then analyzed using Origin Lab software. For each sequence, two fluorescence peaks and the corresponding temperatures were identified: one corresponding to the triplex system and the other to the duplex system. The ratios of the fluorescence value (y) at the triplex and duplex melting temperatures at different TFO concentrations were then plotted against the logarithm of TFO concentration (x). These values were then fitted using the same dose-response function as the one in the previous paragraph.

# ***In Vitro* Transcription**

RNA polymerization experiments were carried out using a Victor X4 Multilable Micro plate reader. Samples were prepared first to contain only 100nM of the double strand transcription unit, specific concentrations of the selected TFO, modified NEB buffer and ultrapure water. Then they were put at +4°C for a 30-minute incubation. Immediately after the incubation, 4 mM of rNTPs, 160 µM of DFHBI-1T and 16U/mL of RNA polymerase holoenzyme were added to the samples. All the calculations were made to have a final volume of 25 µL in each sample. The samples were then transferred to a Lumox 384 multiwell plate, which was covered with a transparent seal. The plate was then loaded in the plate reader set at 29°C for an 8-hour fluorescence signal collection. The excitation wavelength was 485 nm, and the fluorescence signal was filtered at 535 nm. Time-dependent fluorescence signals were then analyzed using Origin Lab software, where the rates for each sample were fitted linearly in a two-hour range. Then, the rates were normalized with respect to the control rate value, and they were plotted in a bar graph.

# ***In Vitro* Transcription-Translation**

Transcription-translation experiments were performed similarly to RNA polymerization experiments. The templates used in this case were prepared by annealing the different promoting sequences to the super-folder Green Fluorescent Protein (sfGFP) codifying DNA strand, which was performed with a simple PCR. Four different FWD primers for sfGFP were purchased, each one carrying one bacterial promoting sequence of interest, i.e., sraGp, poxBp1, and safAp. Only one RVS primer for sfGFP was purchased, which contained the polymerization stop sequence. The PCR cycles were performed using the commercial kit One Taq 2X Master Mix with Standard Buffer with the following quantities: 25 µL of Master Mix, 200 nM of FWD primer, 200 nM of RVS primer, and 0.5 nM of the sfGFP template. The polymerization reaction was carried out in the Mastercycler with the thermocycling conditions for a routine PCR recommended in the commercial kit instructions (20 cycles). The obtained elongated sfGFP template (i.e., added with the transcription promoting sequence and the stop sequence) was evaluated via electrophoresis, and its concentration for all the produced templates was defined as 100 nM (figure s10). The elongated sfGFP templates were stored at -20°C and used for the second part of the experiment.

For the actual transcription-translation experiments, the first set of samples was prepared to contain 50 nM of the elongated sfGFP template, 500 nM of the corresponding TFO, modified NEB buffer and ultrapure water, for a final volume of 25 µL. Samples were then incubated at +4°C for 30 minutes. The second set of samples was prepared to contain 3 µL of NEBExpress S30 synthesis extract, protein synthesis buffer 0.5X, 400U mL^-1^ of­­­­­ RNase inhibitors and 5 µL of the first set of samples. These samples were then transferred in a Lumox 384 multiwell plate, which was covered with a transparent seal. The plate was loaded in Victor X4 plate reader, set at 29°C, for an 8-hour fluorescence signal collection in linear shaking conditions. The excitation wavelength was 390 nm and the fluorescence signal was filtered at 535 nm. Time-dependent fluorescence signals were then analyzed using Origin Lab software, where the rates for each sample were fitted linearly in a two-hour range. Then, the rates were normalized with respect to the control rate value, and they were plotted in a bar graph.

# **PCR product quantification**

An electrophoretic gel was employed to estimate the concentration of the primer-elongated GFP template obtained by PCR (Figure s2, left panel). The quantification of the PCR product concentrations was based on the RVS primer amount that did not anneal with the template. To do so, an RVS primer calibration curve was built in the same electrophoretic gel (Figure s2, right panel).

The quantification estimation is here summarized. From the calibration curve, the amount of not-annealed RVS primer after the PCR protocol was determined as 1.0 ng. By knowing the volume used in the well (*i.e.*, 10 µL) and the MW (*i.e.*, 17763.5 g/mol), the remaining RVS primer concentration resulted to be 5.6 nM. Since in an ideal PCR protocol the selected template is replicated to reach a concentration almost equal to the initial primer concentrations (each primer concentration used for this PCR protocol was 200 nM), and the remained not-annealed RVS primer concentration was 5.6 nM, then the elongated GFP template final concentration resulted as the difference between the initial and the final RVS primer concentration (namely: 200 nM - 5.6 nM = 194.4 nM final concentration). For simplicity, the final sfGFP template concentration was approximated to 200 nM. Because it is known that the PCR efficiency is not 100%, we can assume an arbitrary yield of 50% due to experimental factors. In the reason of that, the final concentration of the GFP template added with promoter and stop sequences after PCR was defined as 100 nM.


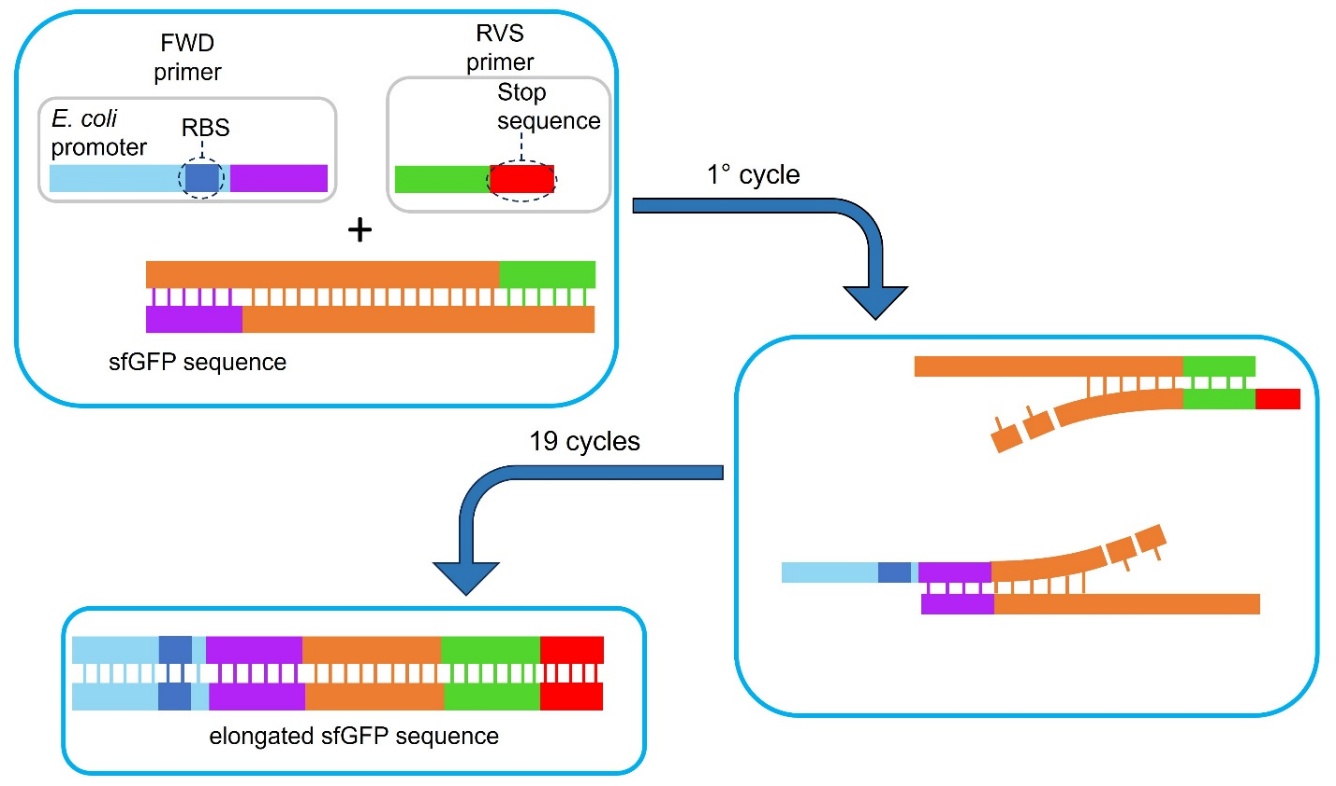


Figure s1. Scheme of the synthesis by PCR of the transcription units used for the in vitro TX-TL experiments.

Figure s2. Electrophoretic gel quality assessment
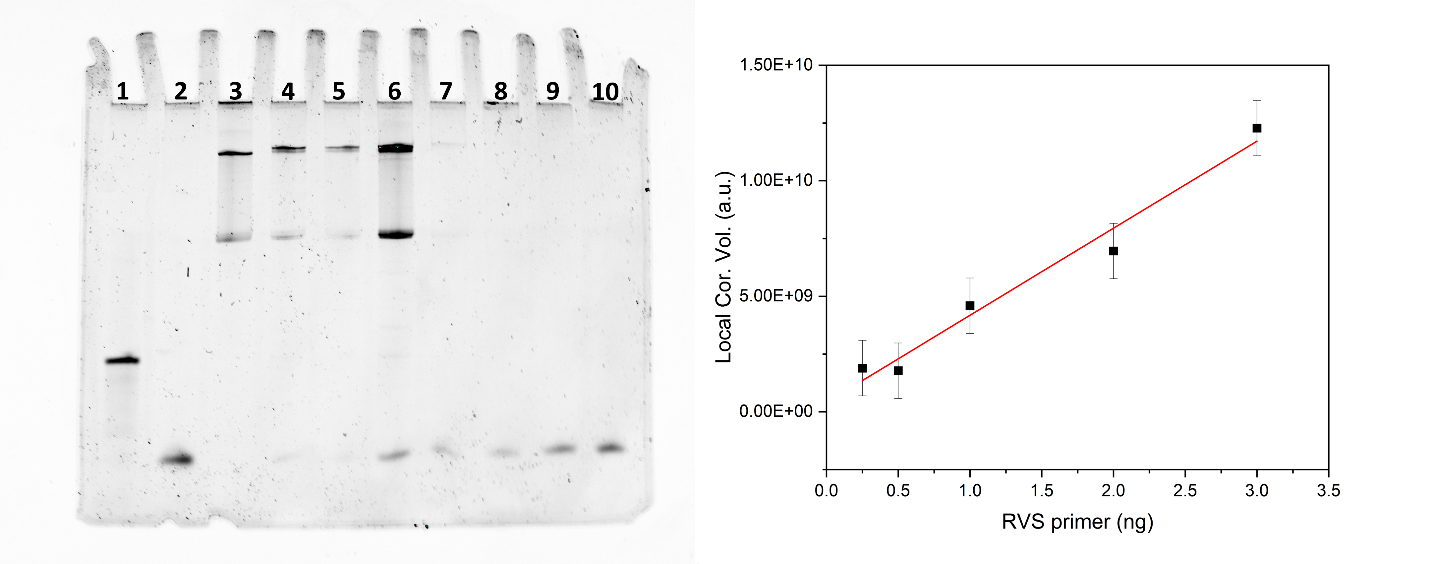
of the PCR-amplified transcription units for sfGFP in vitro transcription-translation. Left panel: Denaturing electrophoretic gel of: 1- FWD primer (5 ng); 2- RVS primer (3 ng); 3- sfGFP sequence (5 ng); 4- PCR product (10 ng); 5- PCR product (5 ng); 6- PCR product (50 ng); 7- RVS primer (0.25 ng); 8- RVS primer (0.5 ng); 9- RVS primer (1 ng); and 10- RVS primer (2ng). Right panel) calibration curve built on the electrophoretic gel band intensities of the RVS primer (i.e., of wells 2, 7, 8, 9, and 10). The calibration curve was used for the quantification of the not-reacted RVS primer present in the PCR products (i.e., detectable in well 6), and then for the indirect estimation of the primer-elongated GFP template (see text). R^2^ = 0.97.

# **Electrophoretic Mobility Shift Assay with RNase H**

Samples were prepared with 100 nM of the double-strand DNA in the presence of 100 mM of the corresponding TFO, in the modified RNAp reaction buffer. After incubating them at 4 °C for 30 min, RNAse H was added at different concentrations (from 0 to 2 units). Samples were then incubated for another 20 minutes at 29°C and electrophoretic mobility shift assay was performed. Runs were conducted in 12% acrylamide/bis-acrylamide gels (19:1) prepared in 1X TBE buffer at pH 6.9, containing 10 mM MgCl_2_, 1.25 mg/mL APS, and 0.05% TEMED. The well loading dye was made with 50% glycerol and 0.1% bromophenol blue. Electrophoresis runs were carried out at 100 V for 2 hours. At the end of the electrophoretic run, the gels were stained in Oxazole Gold 1X staining solution, and emission of the bands was acquired through UV excitation at the iBright1500 imaging system. Results are reported in Figure s6.

# **SDS-PAGE and Western Blot**

The samples used in this experiment are the ones prepared according to our protocol for *in vitro* transcription-translation, without performing GFP signal reading. Instead, samples were incubated at 29°C for 8 hours in a thermocycler and then stored at -80°C until the electrophoretic run was performed. EMSA runs were conducted in a 5% acrylamide/bis-acrylamide (29:1) stacking gel, containing 0.13 M Tris (pH 6.8), 0.1% SDS, 0.1% APS, and 0.12% TEMED, and in a 12% acrylamide/bis-acrylamide (29:1) running gel, containing 0.375 M Tris (pH 8.8), 0.1% SDS, 0.1% APS, and 0.04% TEMED. Before the run, Bradford assay was performed to state the amount of proteins contained in each sample, and absorbance was measured with a Cary 60 UV-Visible spectrophotometer. Loading buffer 1X (0.1M DTT, 0.05 M Tris, pH 6.8, 5% glycerol and 0.02% bromophenol blue) was added to a sample volume corresponding to 15 µg of total protein content, for a final concentration of 0.5X, and they were incubated at 100°C for 5 minutes. SDS-PAGE run was performed at a constant current of 12 mA for 30 min and then the current was set to 24 mA for 2 hours. The transfer proteins for the polyacrylamide gel to the appropriate membrane was done through a Trans-Blot Turbo Transfer System by Bio-Rad using Trans-Blot Turbo equipment (BIORAD, Hercules, USA). The transfer was done using the program “mixed molecular weights”, run at constant current of 1.3 A for 7 minutes. The membrane was then incubated on a shaker overnight at 4°C with 15 mL of saturation buffer, constituted by 0.05 M Tris, 0.15 M, NaCl and 3% BSA, at a pH of 7.5. As a second step, the membrane was incubated at room temperature on a shaker for 2.5 hours in a solution containing anti-sfGFP polyclonal primary antibody (1 mg/mL) diluted 1:1000 in 1% BSA, 0.05 M Tris, 0.15 M NaCl, and 0.01% sodium azide, at a pH of 7.5. Three washes of 10 minutes each were done after the incubation, with a solution containing 0.05 M Tris, 0.15 M NaCl, and tween 20 (1.5 mL for 1 L of final volume), at a pH of 7.5. Secondly, the membrane was incubated for another 2.5 hours on a shaker with a solution containing the Goat anti-rabbit IgG secondary antibody (0.8 mg/mL) diluted 1:10000 in 1% BSA, 0.05 M Tris, and 0.15 M NaCl, at a pH of 7.5. Another three washes were performed in the same way as in the previous step. Finally, Clarity Western ECL Substrate (chemiluminescence method) was added to the surface of the membrane, and after one minute the band image was acquired at the iBright1500 imaging system.

# **DNA-RNA triplex modelling using 3D-NuS and extraction of structural distances**

To generate models of the studied DNA-DNA-RNA system, the web server 3D-Nus (3-Dimensional Nucleic Acid Structures) was used. By selecting the ‘Triplex’ tool, it is possible to generate models of intermolecular triplex structures. For each promoter sequence, the ‘triplex sub-class’ was set as ‘DDR’ and the TTS strands were included as ‘first’ and ‘second sequence’ in the corresponding box. As the ‘third sequence’, the relative TFO, either purinic or pyrimidinic, was inserted. As only some triplex configurations are allowed by the server, for the purinic TFO the ‘triplex class’ was set as ‘anti-parallel’, while for the pyrimidinic TFO the class was set as ‘parallel’. After submitting the sequences, the server generated a 3D model, which was then exported as a PDB file. PDB files generated by the software are available online at… To extract structural distances and lengths of the nucleic acid structures, the online tool MolStar 3D viewer from RCSB PDB (Research Collaboratory for Structural Bioinformatics Protein Data Bank) was used. Several measurements were acquired for each triplex system, and the mean was then calculated.

# **Biphasic gaussian fitting of the triplex-induced distortion-modulation effect**

The experimental transcription modulation percentages (y) for the different analyzed triplexes were plotted against the average distance between nucleotide pairs belonging to the same plane of the Watson-Crick/Hoogsteen interactions at the 3’ of the polypurinic sequence (x). These experimental points were fitted using a custom peaked biphasic sigmoidal function:

$$y=d+\frac{c-d}{1+{10}^{(xc-x)p}}+ \frac{c-d+b x}{1+ {10}^{{(b\left( x-xc \right))}^{2}}}$$

Where d and c are the left and right asymptotes, respectively, x_c_ is the peak center, b is related to the height and width of the peak, and p is the slope of the sigmoid at the inflection point.

| Sequence name | 5’-> 3’ sequence |
| --- | --- |
| lrpp | ttgtactaaaaatcgatgttttgctttgacaatcccctggtgttttgcgaaaacattcgaGgaagaaaaaaaacagtattc |
| poxBp1 | ttagtgcctcctttctctcccatcccttccccctccgtcagatgaactaaacttgttaccGttatcacattcaggagatgg |
| safAp | tgcctacagctgtaagaaactccgctcagtactgaagcaccagtcctatttcctcttttcTccagcctgttatattaagca |
| sraGp | cggtgtgttggccgtactggaatttacgaacgatcggattaagcaatgtaatatcctttcTctttcttagacagtacctta |

Table s2. Sequences of the selected E. coli promoter targets. DNA sequences obtained from the screening of E. coli K12 promoter list using Triplexator where TTSs are highlighted in yellow. poxB contains two homopurinic domains but only one was studied as the discarded TTS, highlighted in light blue, was one nucleotide shorter than the minimum length of 15nt. Capitalized nucleotides indicate the +1 position of the promoter.


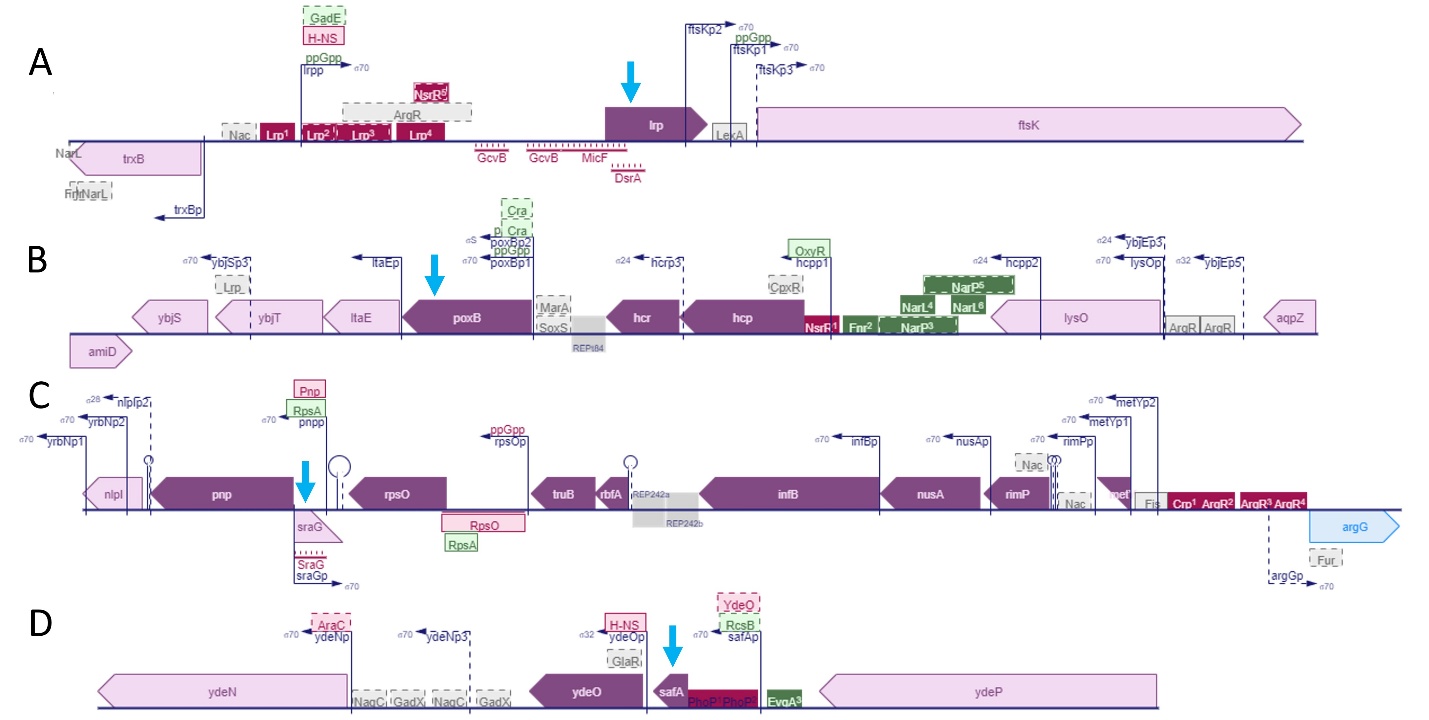


Figure s3. Extended representations of the transcription units associated with the TTS-containing promoters analyzed in this study, which are indicated by light-blue arrows: A- lrpp, B- poxBp1, C- sraGp, and D- safAp. Transcription units are depicted in a wider E. coli K12 genomic context with adjacent genomic elements. Schemes were generated using EcoCyC Genome Browser (<https://biocyc.org/genbro/genbro.shtml?orgid=ECOLI&replicon=COLI-K12>).

| Sequence name | 5’-> 3’ sequence |
| --- | --- |
| lrpp_Pu | aaaaaaaagaaggag |
| lrpp_Py | cuccuucuuuuuuuu |
| poxBp1_Pu | ggaggaaagagaggg |
| poxBp1_Py | cccucucuuuccucc |
| safAp_Pu | aaaggagaaaagagg |
| safAp_Py | ccucuuuucuccuuu |
| sraGp_Pu | aggaaagagaaagaa |
| sraGp_Py | uucuuucucuuuccu |

Table s3. RNA TFOs: Sequence names indicate the target (lrpp, poxBp1, safAp, or sraGp) and the triplex motif (Pu= purinic or Py= pyrimidinic) generated by association of the TFO with the respective dsDNA.

| Sequence name | 5’-> 3’ sequence |
| --- | --- |
| lrpp(+) | gaggaagaaaaaaaa |
| Lrpp(-) | ttttttttcttcctc |
| poxBp1(+) | cctcctttctctccc |
| poxBp1(-) | gggagagaaaggagg |
| safAp(+) | tttcctcttttctcc |
| safAp(-) | ggagaaaagaggaaa |
| sraGp(+) | tcctttctctttctt |
| sraGp(-) | aagaaagagaaagga |

Table s4. TTS sequences excised from promoter sequences reported in Table s1.

**
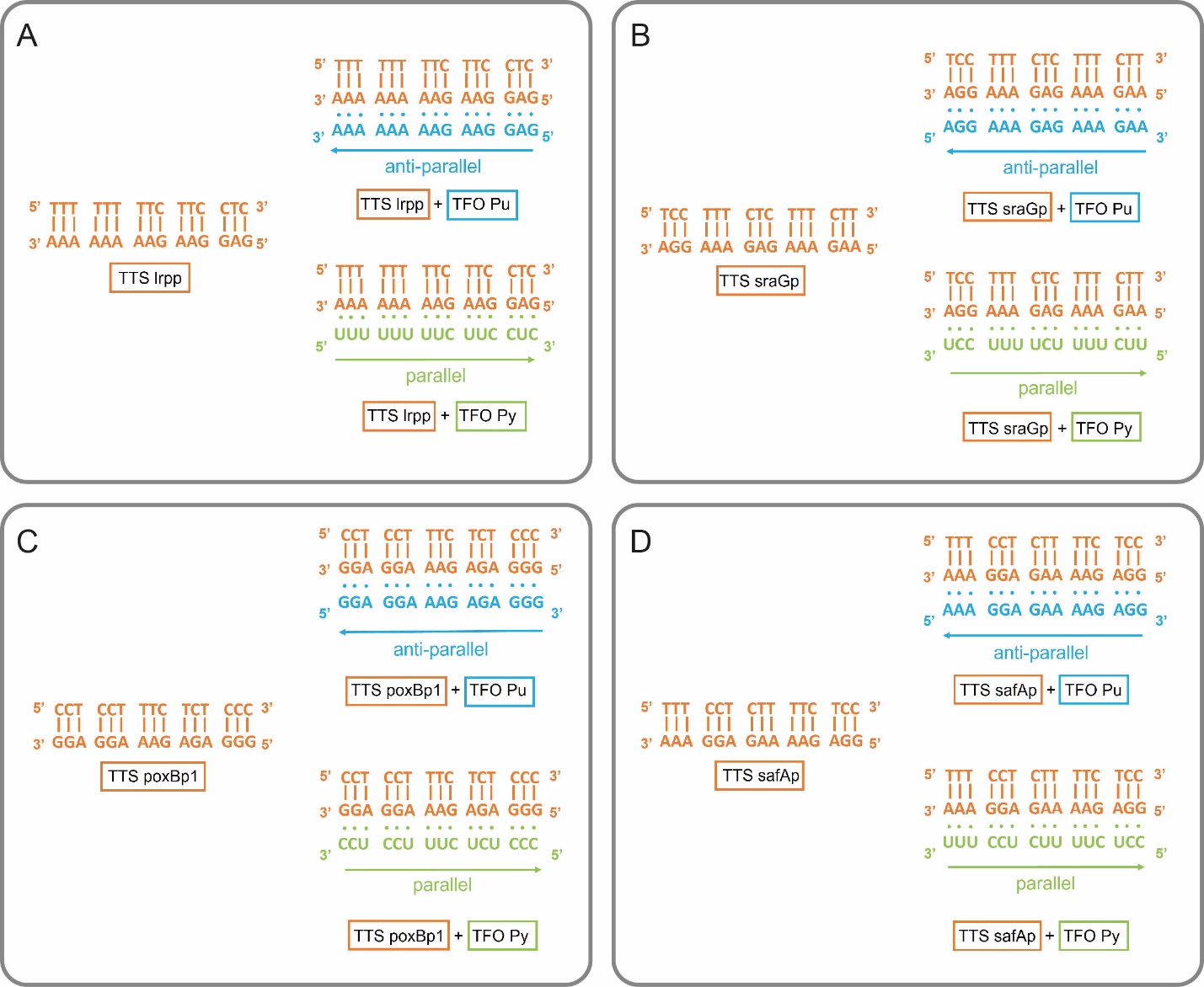
**

Figure s4. Schematic representation of the triplex geometries associated with the structures characterized in this study.


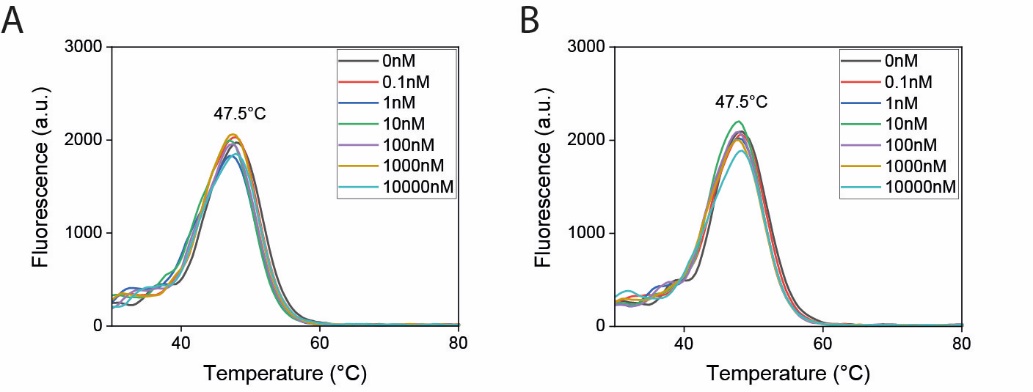


Figure s5. Melting curves of A- lrpp Pu and B- lrpp Py.

| Sequence name | 5’-> 3’ sequence |
| --- | --- |
| sraGp mismatch(+) | tcctttcgctttctt |
| sraGp mismatch(-) | aagaaagcgaaagga |

Table s5. Modified sequences to generate a sraGp TTS DNA duplex that comprises a mismatch (highlighted in yellow) with its corresponding purinic or pyrimidinic TFOs.

*
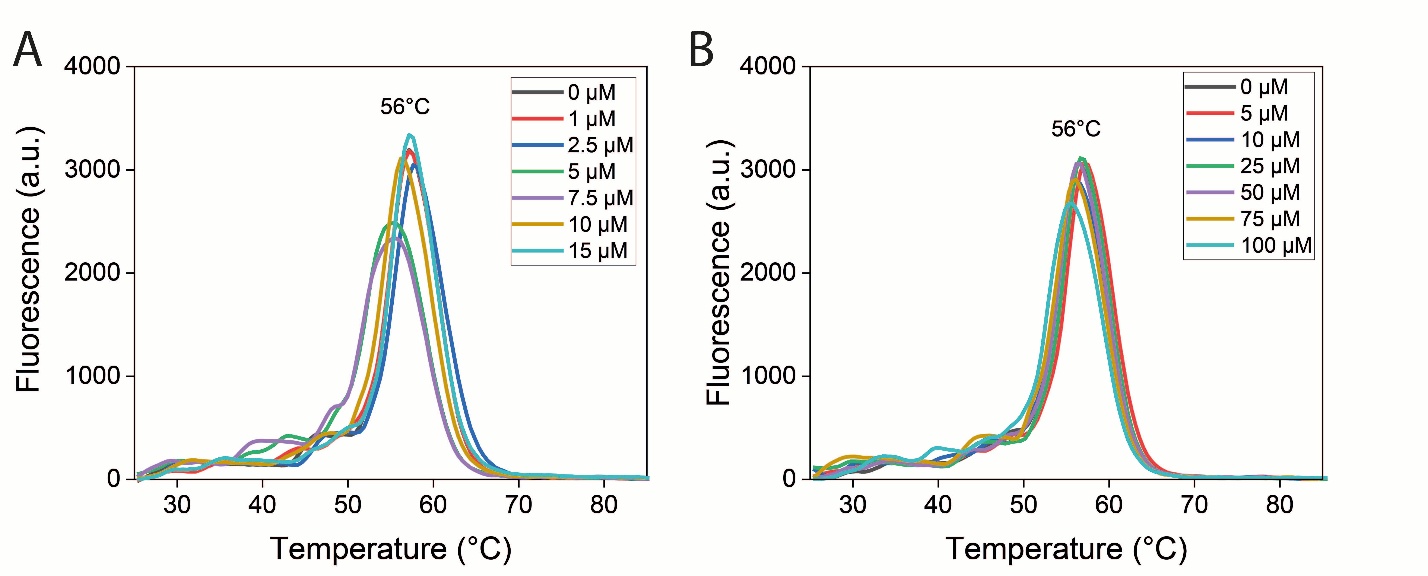
*

Figure s6. Melting curves of sraGp mismatch-TTS with increasing concentration of corresponding A- purinic TFO, and B- pyrimidinic TFO. Only a single peak is visible, indicating only one dissociation event, i.e. the duplex dissociation.


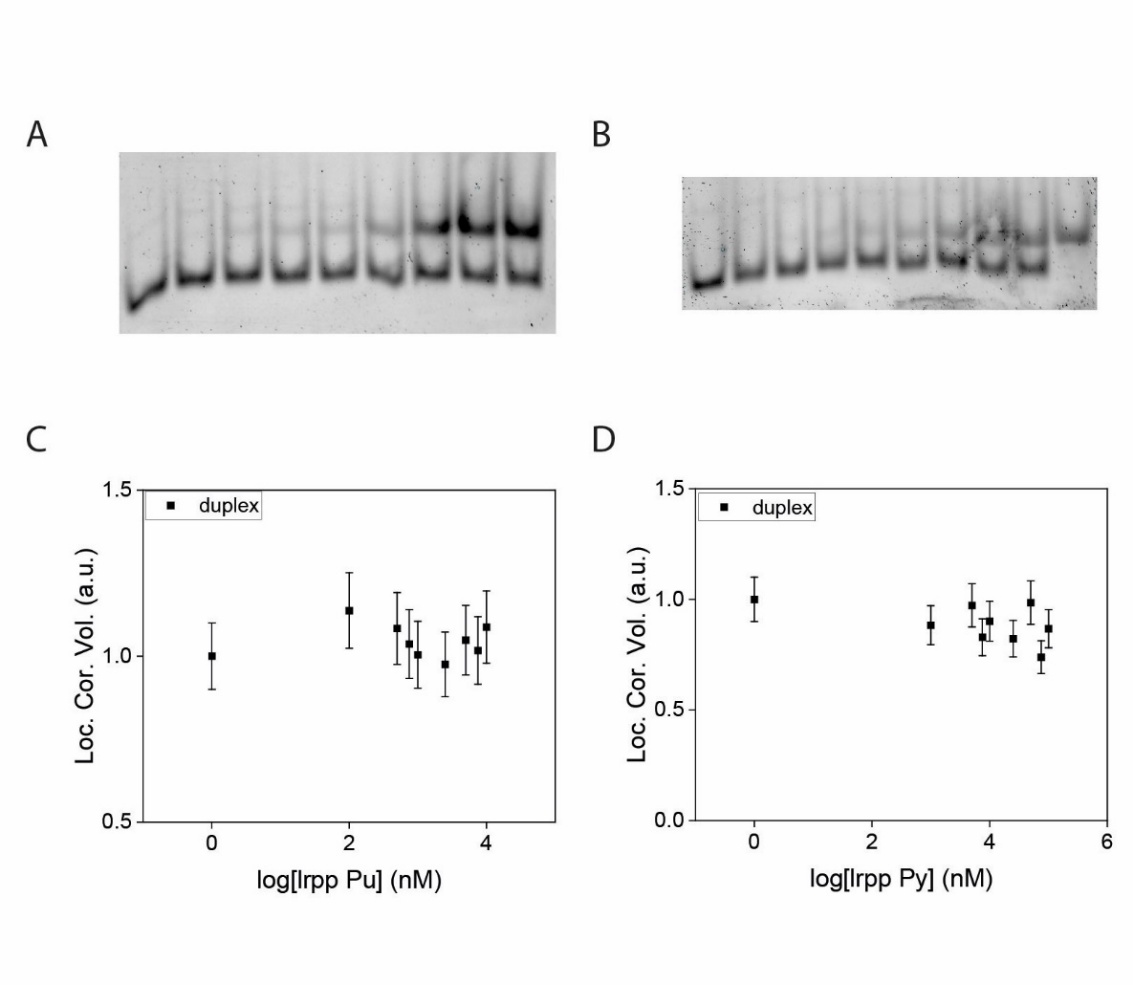


Figure s7. Electrophoretic mobility shift assay (EMSA) and band analysis of duplex bands. A, B, C, and D – EMSA and band analysis of lrpp TTS in the presence of different concentrations of the appropriate TFO.

| Sequence name | 5’-> 3’ sequence |
| --- | --- |
| sraGp Pu complementary | ttctttctctttcct |
| sraGp Py complementary | aggaaagagaaagaa |

Table s6. sraGp DNA sequence complementary to either its corresponding purinic (Pu) or pyrimidinic (Py) TFO to form RNA-DNA heteroduplexes used in the positive RNAse H control.


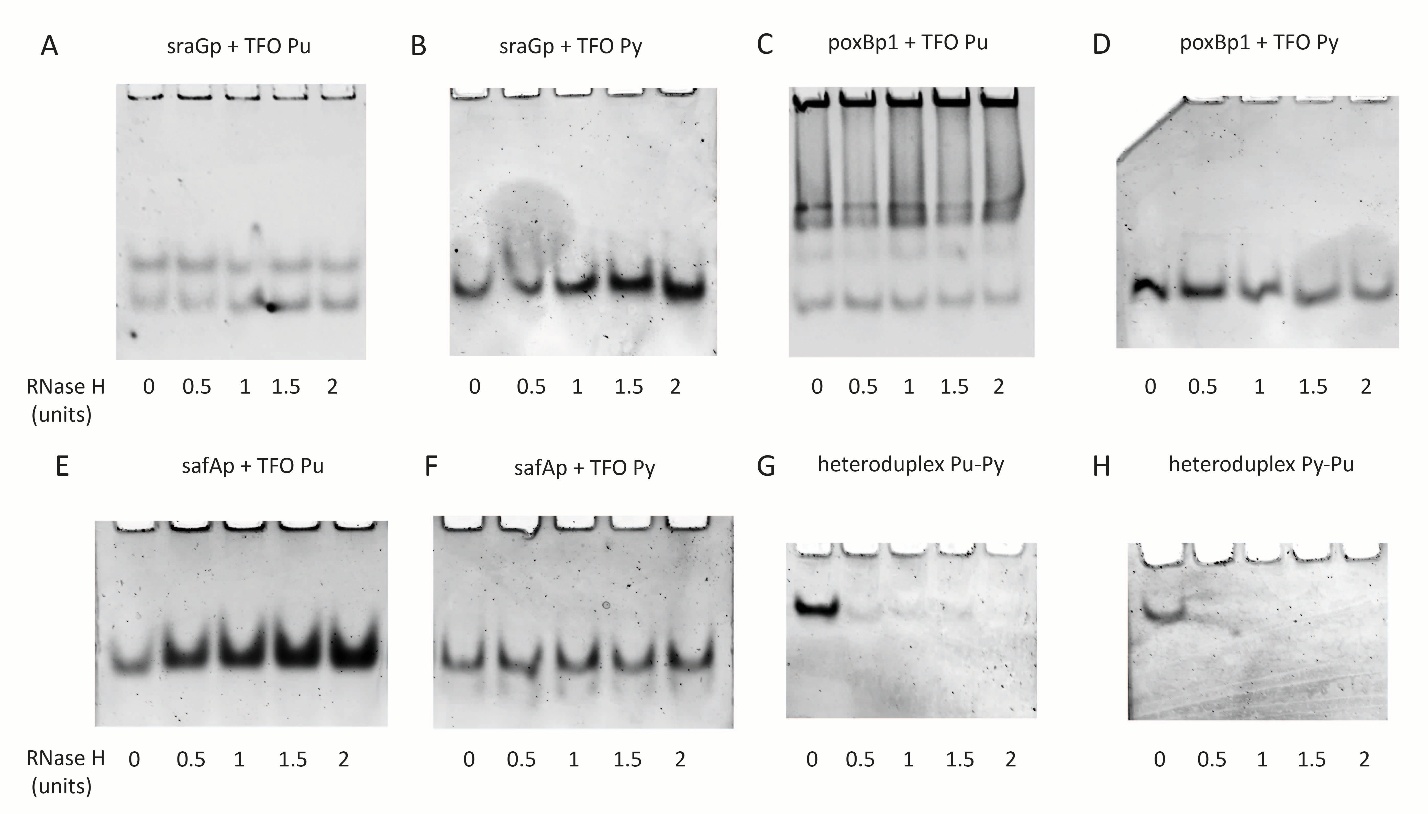


Figure s8. Electrophoretic mobility shift assay (EMSA) of: A, B- sraGp; C, D- poxBp1; and E, F- safAp TTSs with their corresponding purinic (A, C, E) or pyrimidinic (B, D, F) TFO. G, and H- EMSA of heteroduplexes formed by sraGp purinic DNA annealed to its complementary pyrimidinic TFO (G), and by sraGp pyrimidinic DNA annealed to its complementary purinic TFO (H). At the bottom of each panel, the RNase H concentration, in units, is reported.

| TTS | TFO | EMSA Kd (nM) | EMSA  Hill constant | Tm  Hill constant |
| --- | --- | --- | --- | --- |
| safAp | Pu | 60 ± 20 | 2.0 | 1.0 |
|  | Py | 90 ± 30 | 0.9 | 1.3 |
| sraGp | Pu | 80 ± 30 | 0.9 | 2.7 |
|  | Py | 170 ± 60 | 0.2 | 3.5 |
| poxBp1 | Pu | 800 ± 100 | 2.5 | 2.6 |
|  | Py | 10 ± 10 | 9.6 | 1.6 |

Table s7. Dissociation constants of the triplex structures obtained from EMSA experiments and associated Hill constants. Last column: Hill constants obtained from the temperature-dependent triplex/duplex dissociation experiments.


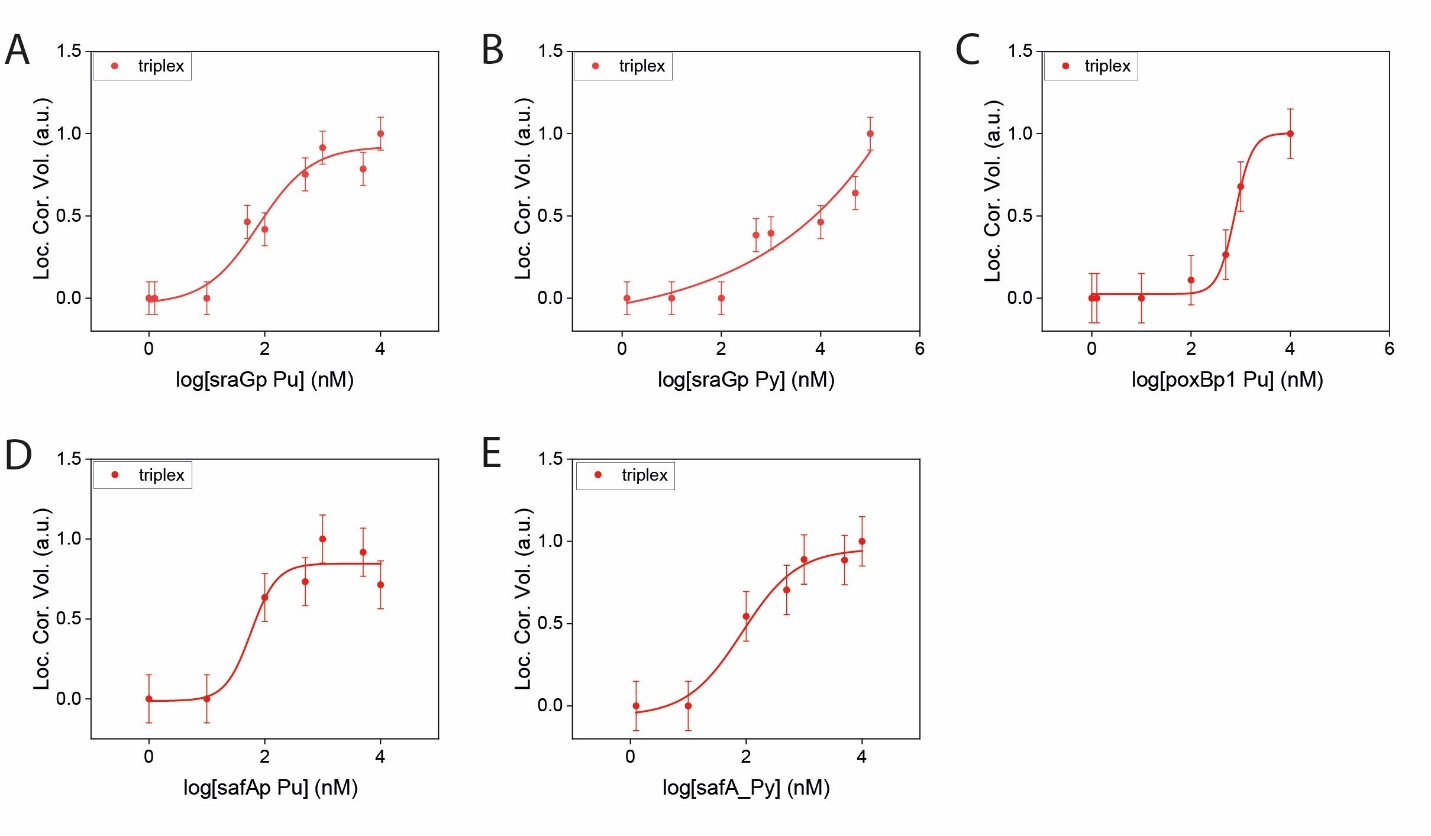


Figure s9. Band analysis of triplex bands from electrophoretic mobility shift assay. A and B - Analysis of sraGp; C - analysis of poxBp1 Pu; D and E- analysis of safAp.

| Sequence long name | Transcription units (yellow-highlighted parts in the (+) strand correspond to triplex target domains); all sequences are 5’->3’. |
| --- | --- |
| 1_sraGp(+) | TTGACA TCCTCTTCTCCTCC TATAAT tcctttcTctttctt GGAACGAGACGGTCGGGTCCAGATATTCGTATCTGTCGAGTAGAGTGTGGGCTCGTTCC |
| 1*_sraGp(-) | GGA ACG AGC CCA CAC TCT ACT CGA CAG ATA CGA ATA TCT GGA CCC GAC CGT CTC GTT CCA AGA AAG AGA AAG GAA TTA TAG GAG GAG AAG AGG ATG TCA A |
| 2_sraGp(+) | TTGACA TCCTCTTCTCCTCC TATAAT aagaaagagaaagga GGAACGAGACGGTCGGGTCCAGATATTCGTATCTGTCGAGTAGAGTGTGGGCTCGTTCC |
| 2*_sraGp(-) | GGA ACG AGC CCA CAC TCT ACT CGA CAG ATA CGA ATA TCT GGA CCC GAC CGT CTC GTT CCT CCT TTC TCT TTC TTA TTA TAG GAG GAG AAG AGG ATG TCA A |
| 3_sraGp(+) | TTGACA tcctttcTctttctt TATAAT AAAAAAA GGAACGAGACGGTCGGGTCCAGATATTCGTATCTGTCGAGTAGAGTGTGGGCTCGTTCC |
| 3*_sraGp(-) | GGA ACG AGC CCA CAC TCT ACT CGA CAG ATA CGA ATA TCT GGA CCC GAC CGT CTC GTT CCT TTT TTT ATT ATA AAG AAA GAG AAA GGA TGT CAA |
| 4_sraGp(+) | TTGACA aagaaagagaaagga TATAAT AAAAAAA GGAACGAGACGGTCGGGTCCAGATATTCGTATCTGTCGAGTAGAGTGTGGGCTCGTTCC |
| 4*_sraGp(-) | GGA ACG AGC CCA CAC TCT ACT CGA CAG ATA CGA ATA TCT GGA CCC GAC CGT CTC GTT CCT TTT TTT ATT ATA TCC TTT CTC TTT CTT TGT CAA |
| 5_poxBp1(+) | TTGACA TCCTCTTCTCCTCC TATAAT cctcctttctctccc GGAACGAGACGGTCGGGTCCAGATATTCGTATCTGTCGAGTAGAGTGTGGGCTCGTTCC |
| 5*_poxBp1(-) | GGA ACG AGC CCA CAC TCT ACT CGA CAG ATA CGA ATA TCT GGA CCC GAC CGT CTC GTT CCG GGA GAG AAA GGA GGA TTA TAG GAG GAG AAG AGG ATG TCA A |
| 6_poxBp1(+) | TTGACA TCCTCTTCTCCTCC TATAAT gggagagaaaggagg GGAACGAGACGGTCGGGTCCAGATATTCGTATCTGTCGAGTAGAGTGTGGGCTCGTTCC |
| 6*_poxBp1(-) | GGA ACG AGC CCA CAC TCT ACT CGA CAG ATA CGA ATA TCT GGA CCC GAC CGT CTC GTT CCC CTC CTT TCT CTC CCA TTA TAG GAG GAG AAG AGG ATG TCA A |
| 7_poxBp1(+) | TTGACA cctcctttctctccc TATAAT AAAAAAA GGAACGAGACGGTCGGGTCCAGATATTCGTATCTGTCGAGTAGAGTGTGGGCTCGTTCC |
| 7*_poxBp1(-) | GGA ACG AGC CCA CAC TCT ACT CGA CAG ATA CGA ATA TCT GGA CCC GAC CGT CTC GTT CCT TTT TTT ATT ATA GGG AGA GAA AGG AGG TGT CAA |
| 8_poxBp1(+) | TTGACA gggagagaaaggagg TATAAT AAAAAAA GGAACGAGACGGTCGGGTCCAGATATTCGTATCTGTCGAGTAGAGTGTGGGCTCGTTCC |
| 8*_poxBp1(-) | GGA ACG AGC CCA CAC TCT ACT CGA CAG ATA CGA ATA TCT GGA CCC GAC CGT CTC GTT CCT TTT TTT ATT ATA CCT CCT TTC TCT CCC TGT CAA |
| 9_safAp(+) | TTGACA TCCTCTTCTCCTCC TATAAT tttcctcttttcTcc GGAACGAGACGGTCGGGTCCAGATATTCGTATCTGTCGAGTAGAGTGTGGGCTCGTTCC |
| 9*_safAp(-) | GGA ACG AGC CCA CAC TCT ACT CGA CAG ATA CGA ATA TCT GGA CCC GAC CGT CTC GTT CCG GAG AAA AGA GGA AAA TTA TAG GAG GAG AAG AGG ATG TCA A |
| 10_safAp(+) | TTGACA TCCTCTTCTCCTCC TATAAT ggagaaaagaggaaa GGAACGAGACGGTCGGGTCCAGATATTCGTATCTGTCGAGTAGAGTGTGGGCTCGTTCC |
| 10*_safAp(-) | GGA ACG AGC CCA CAC TCT ACT CGA CAG ATA CGA ATA TCT GGA CCC GAC CGT CTC GTT CCT TTC CTC TTT TCT CCA TTA TAG GAG GAG AAG AGG ATG TCA A |
| 11_safAp(+) | TTGACA tttcctcttttcTcc TATAAT AAAAAAA GGAACGAGACGGTCGGGTCCAGATATTCGTATCTGTCGAGTAGAGTGTGGGCTCGTTCC |
| 11*_safAp(-) | GGA ACG AGC CCA CAC TCT ACT CGA CAG ATA CGA ATA TCT GGA CCC GAC CGT CTC GTT CCT TTT TTT ATT ATA GGA GAA AAG AGG AAA TGT CAA |
| 12_safAp(+) | TTGACA ggagaaaagaggaaa TATAAT AAAAAAA GGAACGAGACGGTCGGGTCCAGATATTCGTATCTGTCGAGTAGAGTGTGGGCTCGTTCC |
| 12*_safAp(-) | GGA ACG AGC CCA CAC TCT ACT CGA CAG ATA CGA ATA TCT GGA CCC GAC CGT CTC GTT CCT TTT TTT ATT ATA TTT CCT CTT TTC TCC TGT CAA |

Table s8. Modelled triplexes. RNA TFOs (first two lines, the purinic TFO assembles in a antiparallel configuration in respect with the polypurinic strand in the duplex DNA, while the pyrimidine TFO assembles parallel to the polypurinic strand in the duplex DNA). DNA duplexes are formed between the “+” and “-” strands (e.g., 1_sraGp duplex is formed between 1_sraGp(+) and 1*_sraGp(-)). Yellow-highlighted portions define the triplex target site.

| Sequence name and molecular weight (MW) | Sequence 5’->3’ |
| --- | --- |
| poxBp1_FWD_  MW 27576 | TAGTGCCTCCTTTCTCTCCCATCCCTTCCCCCTCCGTCAGATGAACTAAACTTGTTACCGAGGAGGACAGCTATGCGTAAAGGCGAGGAG |
| sraGp_FWD_  MW 28031 | TGGCCGTACTGGAATTTACGAACGATCGGATTAAGCAATGTAATATCCTTTCTCTTTCTT AGGAGGACAGCTATGCGTAAAGGCGAGGAG |
| safAp_FWD_  MW 27697 | CTACAGCTGTAAGAA ACTCCGCTCAGTACTGAAGCACCAGTCCTATTTCCTCTTTTCTCC AGGAGGACAGCT ATG CGT AAA GGC GAG GAG |
| RVS  MW 17764 | AAA AGG GGG CCT GAG TGG CCC CTT TTT TTT A TCAT CAT TTG TAC AGT TCA TCC ATA CC |
| sfGFP(+)  MW 221680 | ATGCGTAAAGGCGAGGAGCTGTTCACTGGTGTCGTCCCTATTCTGGTGGAACTGGATGGTGATGTCAACGGTCATAAGTTTTCCGTGCGTGGCGAGGGTGAAGGTGACGCAACTAATGGTAAACTGACGCTGAAGTTCATCTGTACTACTGGTAAACTGCCGGTACCTTGGCCGACTCTGGTAACGACGCTGACTTATGGTGTTCAGTGCTTTGCTCGTTATCCGGACCATATGAAGCAGCATGACTTCTTCAAGTCCGCCATGCCGGAAGGCTATGTGCAGGAACGCACGATTTCCTTTAAGGATGACGGCACGTACAAAACGCGTGCGGAAGTGAAATTTGAAGGCGATACCCTGGTAAACCGCATTGAGCTGAAAGGCATTGACTTTAAAGAAGACGGCAATATCCTGGGCCATAAGCTGGAATACAATTTTAACAGCCACAATGTTTACATCACCGCCGATAAACAAAAAAATGGCATTAAAGCGAATTTTAAAATTCGCCACAACGTGGAGGATGGCAGCGTGCAGCTGGCTGATCACTACCAGCAAAACACTCCAATCGGTGATGGTCCTGTTCTGCTGCCAGACAATCACTATCTGAGCACGCAAAGCGTTCTGTCTAAAGATCCGAACGAGAAACGCGATCATATGGTTCTGCTGGAGTTCGTAACCGCAGCGGGCATCACGCATGGTATGGATGAACTGTACAAATGATGA |
| sfGFP(-)  MW 223071 | TCA TCA TTT GTA CAG TTC ATC CAT ACC ATG CGT GAT GCC CGC TGC GGT TAC GAA CTC CAG CAG AAC CAT ATG ATC GCG TTT CTC GTT CGG ATC TTT AGA CAG AAC GCT TTG CGT GCT CAG ATA GTG ATT GTC TGG CAG CAG AAC AGG ACC ATC ACC GAT TGG AGT GTT TTG CTG GTA GTG ATC AGC CAG CTG CAC GCT GCC ATC CTC CAC GTT GTG GCG AAT TTT AAA ATT CGC TTT AAT GCC ATT TTT TTG TTT ATC GGC GGT GAT GTA AAC ATT GTG GCT GTT AAA ATT GTA TTC CAG CTT ATG GCC CAG GAT ATT GCC GTC TTC TTT AAA GTC AAT GCC TTT CAG CTC AAT GCG GTT TAC CAG GGT ATC GCC TTC AAA TTT CAC TTC CGC ACG CGT TTT GTA CGT GCC GTC ATC CTT AAA GGA AAT CGT GCG TTC CTG CAC ATA GCC TTC CGG CAT GGC GGA CTT GAA GAA GTC ATG CTG CTT CAT ATG GTC CGG ATA ACG AGC AAA GCA CTG AAC ACC ATA AGT CAG CGT CGT TAC CAG AGT CGG CCA AGG TAC CGG CAG TTT ACC AGT AGT ACA GAT GAA CTT CAG CGT CAG TTT ACC ATT AGT TGC GTC ACC TTC ACC CTC GCC ACG CAC GGA AAA CTT ATG ACC GTT GAC ATC ACC ATC CAG TTC CAC CAG AAT AGG GAC GAC ACC AGT GAA CAG CTC CTC GCC TTT ACG CAT |

Table s9. Primer and sfGFP sequences used to produce the TUs for the TX-TL system.


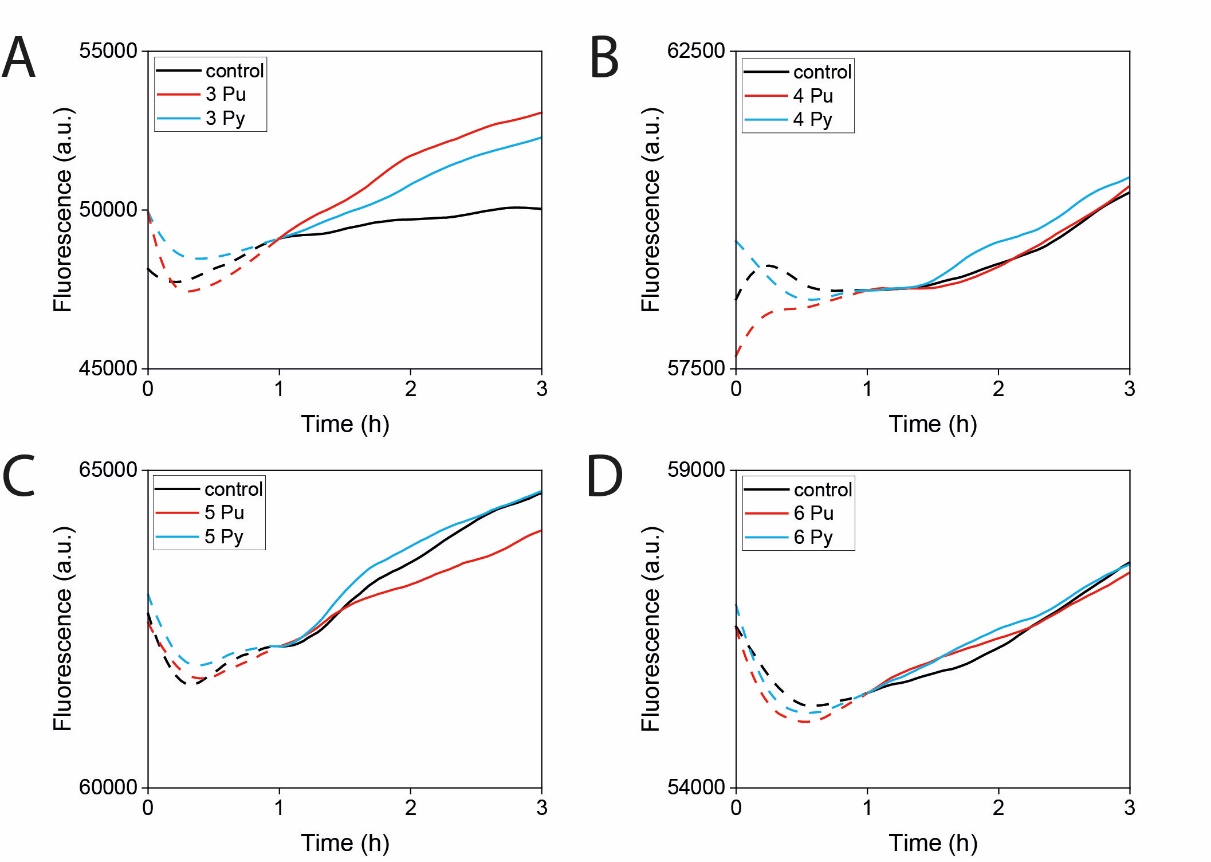


Figure s10. Fluorescence kinetics of transcription in vitro experiments for sraGp template. The dotted part of the experimental data was not used for the linear fitting.


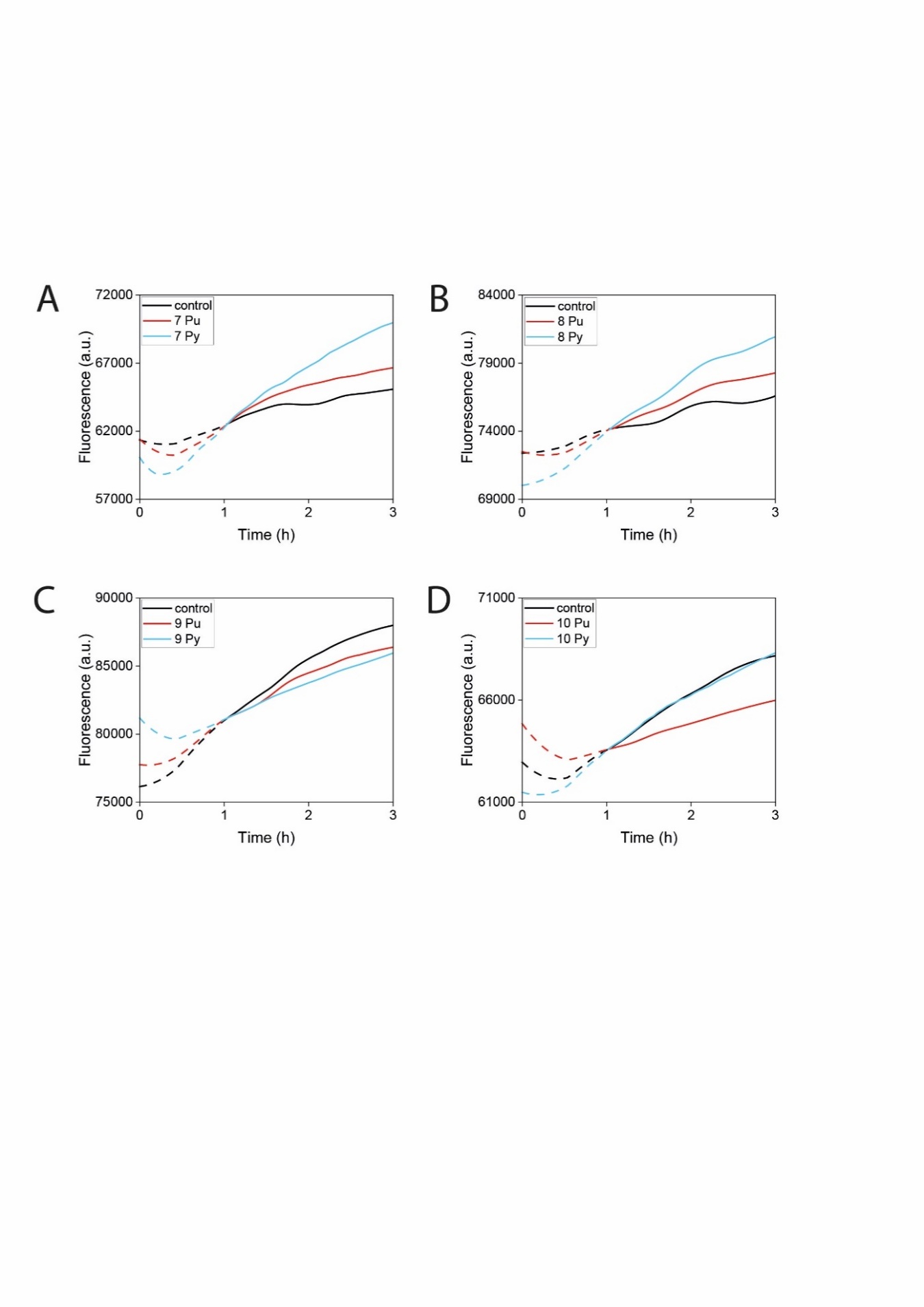


Figure s11. Fluorescence kinetics of transcription in vitro experiments for poxBp1 template. The dotted part of the experimental data was not used for the linear fitting.


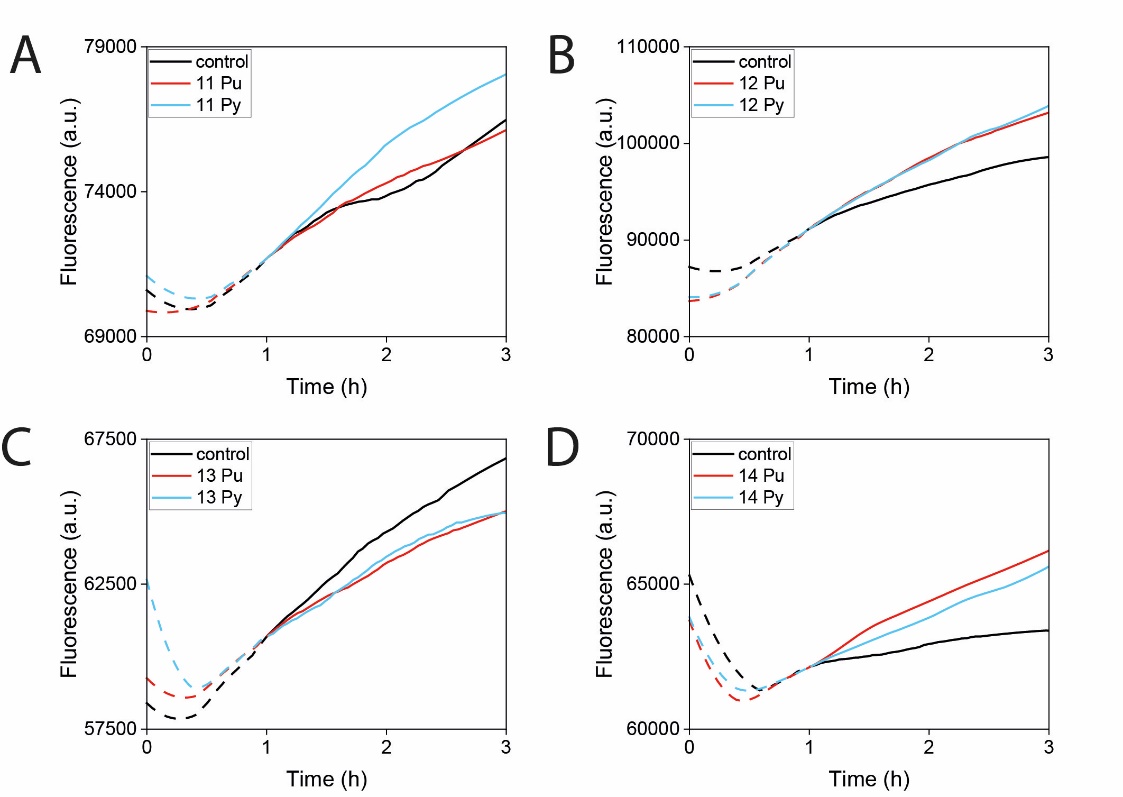


Figure s12. Fluorescence kinetics of transcription in vitro experiments for safA template. The dotted part of the experimental data was not used for the linear fitting.


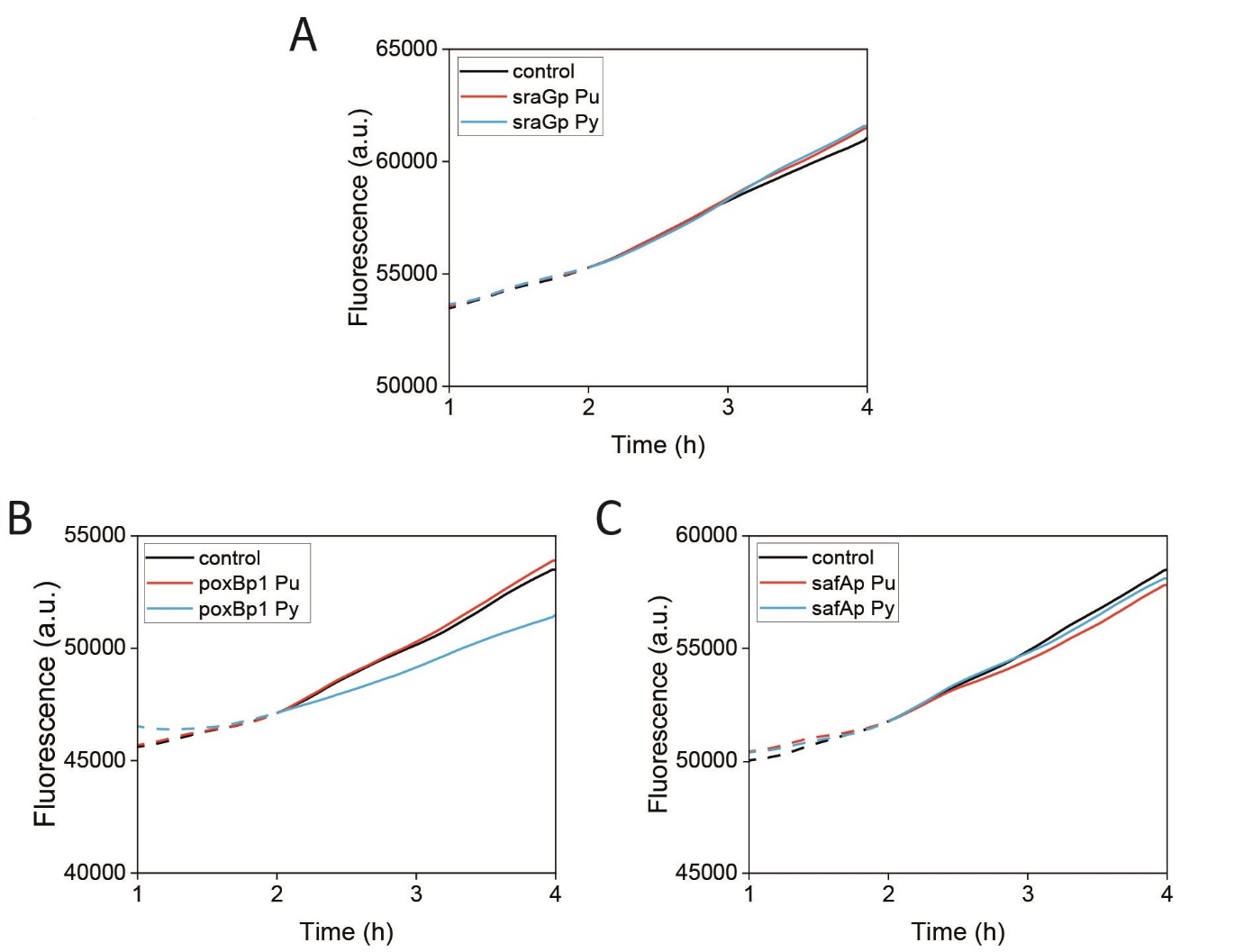


Figure s13. Fluorescence (sfGFP) kinetics of transcription-translation in vitro experiments. A: results for sraGp template, B: results for poxBp1 template, C: results for safAp template. The dotted part of the experimental data was not used for the linear fitting.

*
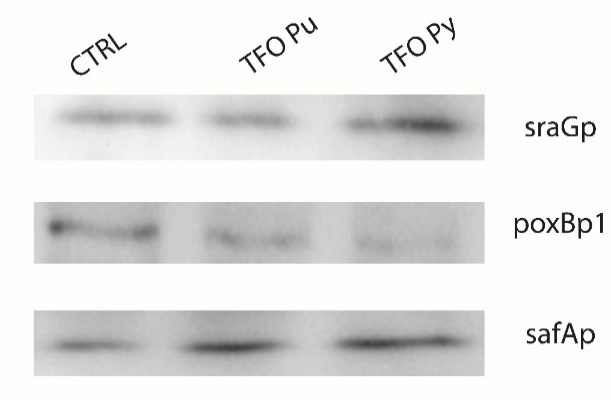
*

Figure s14. sfGFP bands from and exemplary immunoblot analysis.

| TX-TL sequence | TFO | Relative intensity |
| --- | --- | --- |
| sraGp | - | 1 |
|  | Pu | 0.78 |
|  | Py | 1.6 |
| poxBp1 | - | 1 |
|  | Pu | 0.5 |
|  | Py | 0.3 |
| safAp | - | 1 |
|  | Pu | 2.1 |
|  | Py | 2.9 |

Table s10. Band quantification of the blot in Figure s14, obtained as relative intensity.


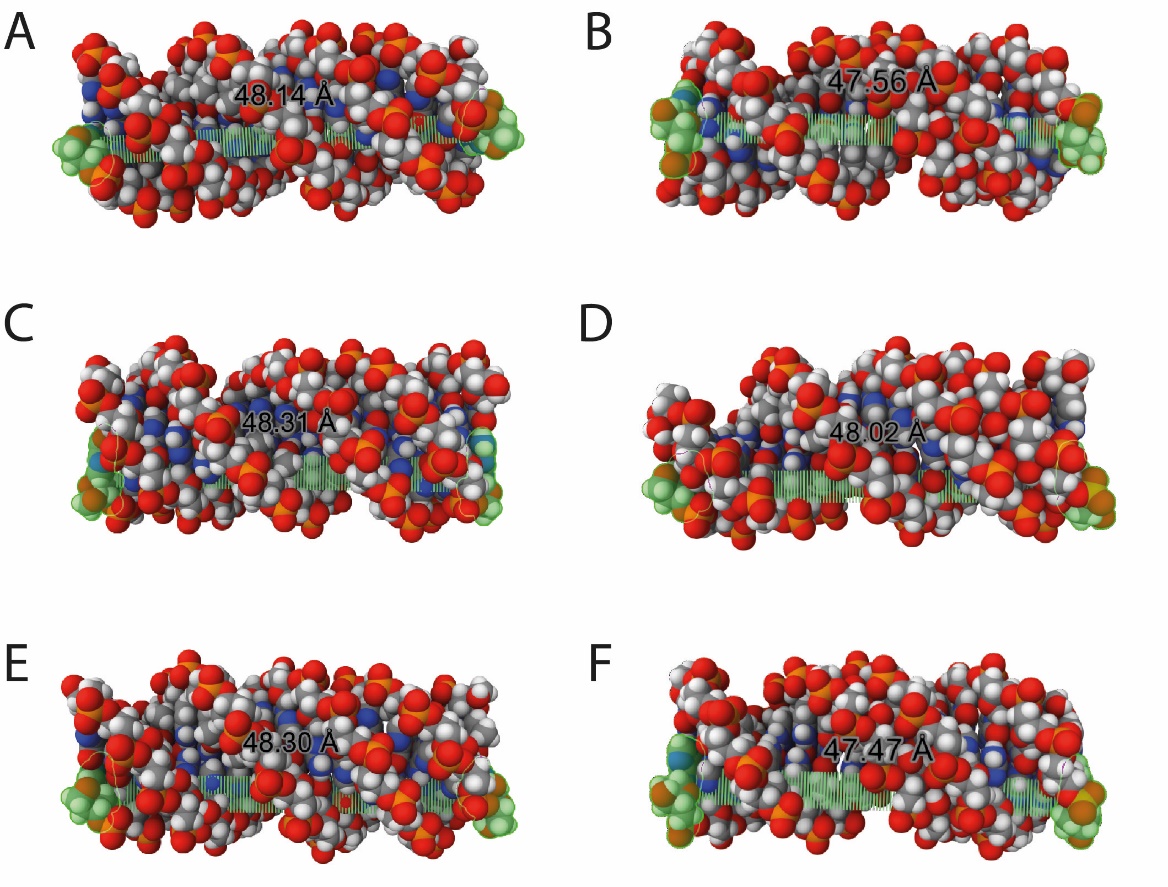


Figure s15. Exemplary view of the molecular models of the triplex complex used for distance measurements. The length is indicated by a green dashed line topped by the length value in Angstrom. On the left part, pyrimidinic motif triplexes for the TTSs found in promoters: B- sraGp, D- poxBp1, F- safAp. On the right part, purine motif triplexes for the TTSs found in promoters: A- sraGp, C- poxBp1, E- safAp.


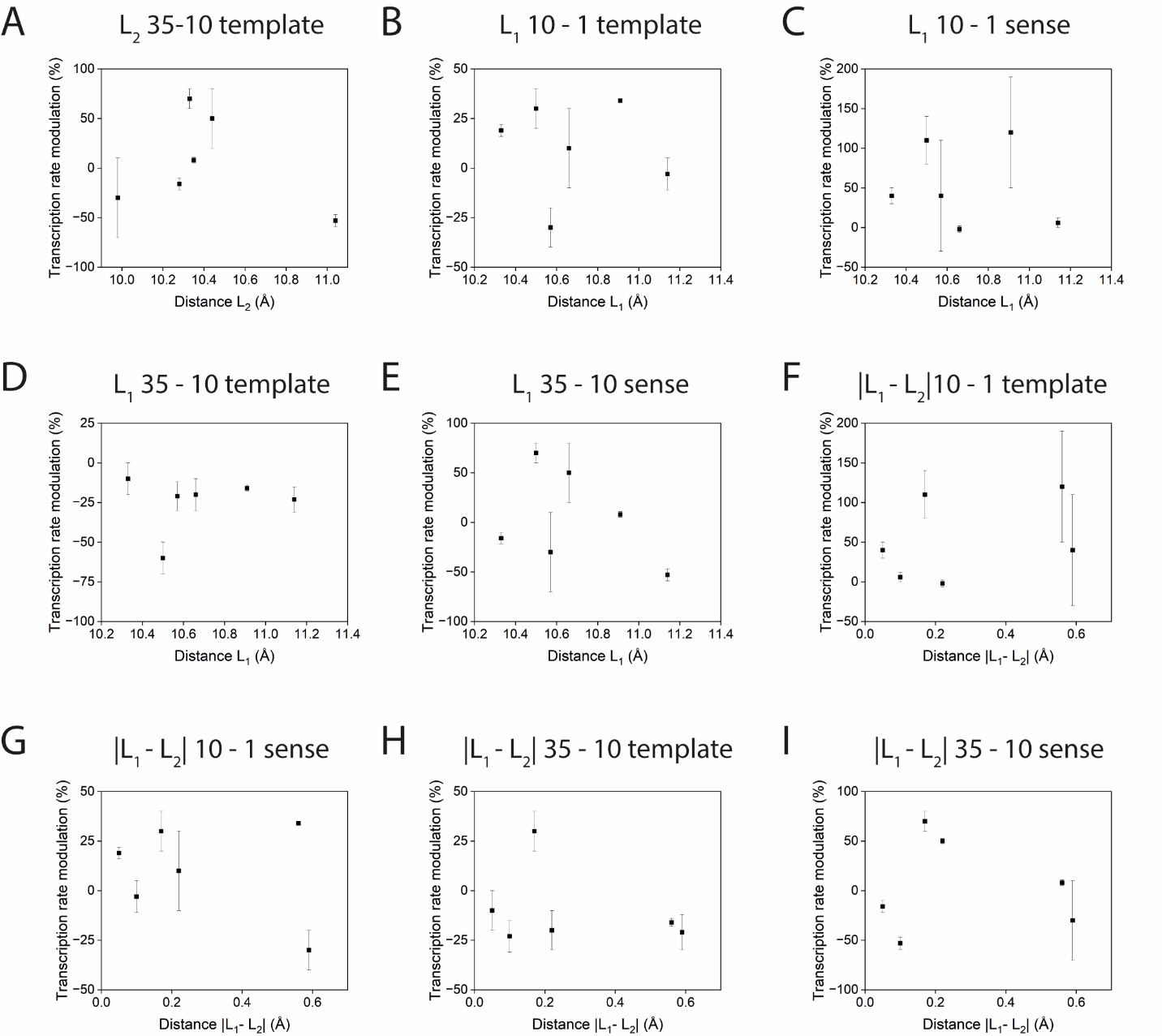


Figure s16. Average nucleotide distances of different triplex geometries plotted against the respective transcription rate modulation. L_1_ and L_2_ are the average distances between nucleotide pairs belonging to the same plane of the Watson-Crick/Hoogsteen interactions at either the 5’ or 3’ of the polypurinic sequence, respectively. |L_1_ and L_2_| is the absolute difference between L_1_ and L_2_. The notation “sense” and “template” refer to the position of the TTS in the triplex geometry, in the sense or the template strand respectively.

| Genome | start position (bp) | end position (bp) | sequence name | strand |
| --- | --- | --- | --- | --- |
| U00096.3 | 912945 | 912957 | Lrpp_anti_Pt | - |
| U00096.3 | 912945 | 912957 | Lrpp_par_Py | + |
| U00096.3 | 3382316 | 3382327 | poxBp1_anti_Pt | - |
| U00096.3 | 3385233 | 3385244 | poxBp1_par_Py | + |
| U00096.3 | 2283961 | 2283972 | safAp_anti_Pt | - |
| U00096.3 | 2283961 | 2283972 | safAp_par_Py | + |
| U00096.3 | 4100296 | 4100308 | sraGp_anti_Pt | - |
| U00096.3 | 4100296 | 4100308 | SraGp_par_Py | + |

Table s11. TFO identification in E. coli str. K-12 substr. MG1655 genome (GenBank: U00096.3). All TFOs were identified once using Blastn search (best match) with associated positions in base pair (bp) in the bacterial genome.
